# Personalized Antibiogram: A Novel Multitask Machine Learning Framework for Simultaneous Prediction of Antimicrobial Resistance Profile With Enhanced Detection of Carbapenem Resistance in Enterobacteriaceae

**DOI:** 10.1093/cid/ciag027

**Published:** 2026-01-17

**Authors:** Michihiko Goto, Anindita Bandyopadhyay, Qianyi Shi, Yaohua Wang, Eli N Perencevich, David Hernandez, W Nick Street

**Affiliations:** Department of Internal Medicine, University of Iowa Carver College of Medicine, Iowa City, Iowa, USA; Center for Access & Delivery Research and Evaluation (CADRE), Iowa City Veterans Affairs Health Care System, Iowa City, Iowa, USA; Department of Internal Medicine, University of Iowa Carver College of Medicine, Iowa City, Iowa, USA; Department of Business Analytics, University of Iowa Tippie College of Business, Iowa City, Iowa, USA; Department of Internal Medicine, University of Iowa Carver College of Medicine, Iowa City, Iowa, USA; Center for Access & Delivery Research and Evaluation (CADRE), Iowa City Veterans Affairs Health Care System, Iowa City, Iowa, USA; Department of Internal Medicine, University of Iowa Carver College of Medicine, Iowa City, Iowa, USA; Center for Access & Delivery Research and Evaluation (CADRE), Iowa City Veterans Affairs Health Care System, Iowa City, Iowa, USA; Department of Internal Medicine, University of Iowa Carver College of Medicine, Iowa City, Iowa, USA; Center for Access & Delivery Research and Evaluation (CADRE), Iowa City Veterans Affairs Health Care System, Iowa City, Iowa, USA; Center for Access & Delivery Research and Evaluation (CADRE), Iowa City Veterans Affairs Health Care System, Iowa City, Iowa, USA; Department of Business Analytics, University of Iowa Tippie College of Business, Iowa City, Iowa, USA

**Keywords:** antimicrobial resistance prediction, machine learning, electronic health record data, Gram-negative rods

## Abstract

**Background:**

Conventional hospital antibiograms summarize aggregated resistance rates, limiting their utility for individualized antimicrobial selection. Existing statistical and machine learning models predict each phenotype separately, ignoring correlations among resistance profiles. We developed novel multitask extreme gradient boosting (XGBoost) models utilizing structured data in electronic health records (EHRs) to predict resistance to 8 antimicrobial classes simultaneously and evaluated their performance within the Veterans Health Administration (VHA).

**Methods:**

We conducted a retrospective multicenter study of *Escherichia coli* and *Klebsiella* spp. isolates collected at 127 hospitals and >1400 clinics from January 2017 to September 2024. Data from January 2017 to September 2023 were used for model development, while data from October 2023 to September 2024 were used for simulated prospective testing. Model performances were compared to hospital antibiograms and single-target XGBoost models.

**Results:**

The training cohort included 536 252 *E. coli* and 246 898 *Klebsiella* spp. isolates; the test cohort included 75 138 and 38 015 isolates, respectively. On the test data, the multitask model achieved overall areas under the receiver operating characteristic curve (AUROCs) of 0.779 (*E. coli*) and 0.810 (*Klebsiella* spp.), with good to excellent per-class performance (AUROC range, 0.743–0.847). A multitask approach improved calibration and decreased false-negative rates for carbapenem resistance while predicting individualized resistance probabilities for all target antimicrobials simultaneously (“personalized antibiograms”).

**Conclusions:**

A multitask XGBoost framework can accurately predict individualized resistance profiles for common Gram-negative pathogens, outperforming conventional antibiograms and single-target models. Personalized antibiograms may enhance the selection of empiric therapy, including the detection of carbapenem resistance in low-endemicity settings.

## Teaser

A multitask XGBoost model using routine electronic health record data simultaneously predicted correlated antimicrobial resistance phenotypes for *E. coli* and *Klebsiella* spp., outperforming conventional antibiograms and single-target models and improving carbapenem resistance detection even in low-endemicity settings.

For decades, hospital antibiograms have served as a tool of empiric antimicrobial decision-making. Aggregated periodically (usually annually), these cumulative susceptibility profiles summarize local resistance patterns by pathogen and are widely distributed to clinicians. However, despite their ubiquity, antibiograms offer limited utility for individualized antimicrobial resistance risk prediction, likely because they do not account for patient-specific factors such as prior antimicrobial exposures, comorbidities, or past microbiological test results [1]. As healthcare moves toward personalized, data-driven care, the limitations of a “one-size-fits-all” resistance summary are increasingly apparent.

Rising resistance across multiple antimicrobial classes in Enterobacterales is a growing public health threat; carbapenem-resistant organisms (CROs) are especially concerning because they markedly restrict therapeutic options and are linked to worse clinical outcomes. The Centers for Disease Control and Prevention (CDC) has designated carbapenem-resistant Enterobacteriaceae (CRE) as an urgent threat [2]. However, their prevalence rates vary geographically and are fortunately still low in many regions of the United States [1, 3, 4]. Conventional hospital antibiograms perform particularly poorly to predict CRO [1], and other approaches to predicting resistance, including rule-based risk scores and statistical models, have had limited success, especially in low-endemicity contexts [5–8]. The infrequent occurrence of CRO cases undermines the performance of conventional regression models and traditional machine learning methods, leading to high false-negative rates and poor calibration [5, 8]. The fundamental methodological challenge lies in the imbalance of the outcome, requiring prediction frameworks that can maximize sensitivity without overly sacrificing specificity or positive predictive value while accommodating the infrequency of the prediction target.

Many existing models treat antimicrobial resistance as a single-label problem, focusing narrowly on each resistance phenotype as an isolated event [5, 8, 9]. This ignores the underlying interdependence among resistance phenotypes. Resistance mechanisms, such as efflux pumps, plasmid-encoded β-lactamases, and porin loss, frequently co-occur and can drive cross-resistance across antimicrobial classes [10]. In particular, resistance to other broad-spectrum beta-lactams is often associated with carbapenem resistance, reflecting shared genetic and environmental risk factors [5, 11]. Furthermore, epidemiologic studies have demonstrated that resistance to nonbeta-lactam antimicrobials also correlates with multidrug-resistant phenotypes and may precede or predict carbapenem resistance in clinical isolates [11, 12].

To address these complexities, we propose a novel, multitask machine learning framework using extreme gradient boosting (XGBoost) to predict resistance across a spectrum of commonly used or clinically important antimicrobials, including carbapenems, penicillins, cephalosporins, fluoroquinolones, and trimethoprim/sulfamethoxazole (TMP/SMX) simultaneously, using routinely available data from electronic health records (EHRs). We selected XGBoost because it is a scalable implementation of gradient boosting that performs well on large, heterogeneous tabular datasets, captures nonlinear effects and interactions, and scales efficiently [13]. By jointly modeling resistance across these agents, our approach captures the latent structure of antimicrobial cross-resistance and enables learning from more prevalent phenotypes to improve the prediction of infrequent but clinically consequential ones (eg, carbapenem resistance). This approach also generates a list of predicted probabilities of resistance for multiple antimicrobials for each organism, presented as a panel (resembling conventional hospital antibiograms in format), while incorporating individualized information (“personalized antibiogram”). Importantly, the multitask formulation itself is model agnostic and could be implemented with alternative ensemble learners in future work [14].

In this work, we aimed to develop and validate novel multitask XGBoost models using structured EHR data to predict antimicrobial resistance profiles, including infrequent but clinically important carbapenem resistance, in the 2 most prevalent Gram-negative organisms in clinical practice, *Escherichia coli* and *Klebsiella* spp., thereby proving the feasibility of personalized antibiograms. We compared the multitask model's performance to conventional hospital antibiograms and standard single-target XGBoost classifiers.

## METHODS

### Study Design and Population

We conducted a retrospective observational study including clinical isolates of *E. coli* and *Klebsiella* spp. reported from 127 hospitals and >1400 clinics located in 48 continental states of the United States within the Veterans Health Administration (VHA) system from January 2017 to September 2024. As a proof-of-concept study, we focused on *E. coli* and *Klebsiella* spp. a priori because these are the most commonly encountered Gram-negative rods (GNRs) in serious GNR infections in community and nosocomial settings [15, 16]. Due to inconsistent reporting of species across sites and years, we excluded *Klebsiella aerogenes* from our *Klebsiella* spp. analysis. Before 2017 (when *K. aerogenes* was reclassified to the genus *Klebsiella* [17]), some facilities reported only *Enterobacter* spp. at the genus level. We used data from 1 January 2017 to 30 September 2023 for model development (training and validation). Data from 1 October 2023 to 30 September 2024 were used to evaluate predictive performance, simulating prospective validation.

Microbiological data for all included isolates, including their susceptibility reports for 8 clinically important antimicrobials for GNR infections (narrow-spectrum [NS] and expanded-spectrum [ES] cephalosporins, aminopenicillins, aminopenicillin/beta-lactamase inhibitor (BLI) [18] combinations, antipseudomonal penicillins/BLI combinations, carbapenems, fluoroquinolones, and TMP/SMX) as well as specimen types were systematically obtained from the Corporate Data Warehouse (CDW), an integrated data repository system from VHA's EHRs. We included all isolates if they had susceptibility reports for at least 1 of 8 target antimicrobial groups. For patients who had included isolates, we also collected demographics (age, gender, and urban/rural status of residential address), comorbidities, preceding procedures within 90 days, past microbiology test results, and past antimicrobial exposures based on pharmacy dispense data at both inpatient and outpatient settings from CDW. We also included facility characteristics and conventional hospital antibiograms from which isolates were reported as predictors. The process for systematically generating hospital antibiograms has been reported elsewhere [1].

The majority of VHA hospitals had on-site microbiology laboratories during the study period. They are required to conduct routine quality control/quality assessment in accordance with the requirements of VHA-designated accreditation organizations (eg, College of American Pathologists) and to use methods and equipment approved by the Food and Drug Administration (FDA) [19].

### Variable Definitions

We categorized antimicrobial agents into 8 groups based on their activities and therapeutic equivalence for Enterobacteriaceae (Supplementary Table 1). If more than 1 agent within a group was tested for the same isolate, we retained the most resistant result. For this study, we used the contemporaneous categorical interpretations (susceptible, intermediate, and resistant) reported by the local laboratories at the time of testing and did not reinterpret minimum inhibitory concentration values. We then recoded these results into a binary outcome, with susceptible coded as 0 and intermediate or resistant combined into a single nonsusceptible category coded as 1 (ie, susceptible vs nonsusceptible). All 8 groups were considered prediction targets for *E. coli*, while we excluded aminopenicillins from targets for *Klebsiella* spp., as they are generally considered intrinsically resistant.

Comorbidities were obtained from inpatient and outpatient diagnoses recorded in EHR as International Classification of Diseases, 10th edition (ICD-10) codes. They were classified into 86 categories based on Hierarchical Condition Categories (HCCs) version 24, developed by the Centers for Medicare & Medicaid Services (CMS) [20]. Procedures were obtained from inpatient and outpatient procedure records recorded as ICD-10 procedure codes or Current Procedural Terminology (CPT) codes. Those codes were classified into 224 categories based on the Clinical Classifications Software (CCS) developed by the Agency for Healthcare Research and Quality [21].

For patients with prior isolates of the same organism, susceptibility reports for the most recent isolate and the time elapsed since that isolate were used as predictors; no prior isolate was treated as a distinct value. Past isolate data within 72 hours before specimen sampling was excluded, acknowledging the typical 2–3 day turnaround for susceptibility test results. Past antimicrobial exposures were categorized into time intervals (0–3, 4–7, 8–14, 15–30, 31–90, 91–365, and >365 days), with the number of unique exposure days recorded for each antimicrobial group, or zero if no exposure occurred.

### Machine Learning Model Development

We built a single XGBoost model for each organism that predicts resistance to multiple antimicrobials simultaneously by reorganizing the data so that each line represents a single culture–antimicrobial pair. Instead of training a separate model for each antimicrobial class, we fit a single unified model with shared predictors and an indicator of the antimicrobial class to which each row pertains. This multitask formulation enables the model to learn patterns that generalize across drugs while capturing drug-specific differences.

During training, we used 5-fold cross-validation, keeping all samples from the same patient in the same fold and excluding calendar year as a predictor to reduce information leakage. For comparison, we also fit (1) a logistic regression model for each antimicrobial class as an additional comparison, using a conventional hospital antibiogram as the sole input variable, following a published protocol [1], and (2) single-task XGBoost models separately for each antimicrobial class.

Details of the model development process are provided in the Technical Supplement. All model developments and computations were performed on Python version 3.10.9 (Python Software Foundation, Wilmington, Delaware) and XGBoost 2.0.3 (Distributed Machine Learning Community, University of Washington). We followed the Transparent Reporting of a multivariable prediction model for Individual Prognosis or Diagnosis + Artificial Intelligence (TRIPOD+AI) statement for reporting [22].

### Model Evaluation and Sensitivity/Subgroup Analyses

We evaluated conventional hospital antibiogram-based logistic regression, single-task XGBoost, and unified multitask XGBoost models on the test set. Performance was assessed per antimicrobial and overall using the area under the receiver operating characteristic curve (AUROC), the area under the precision–recall curve (AUPRC), and the Brier score, with Brier skill defined relative to the logistic regression antibiogram baseline [23]. We interpreted Brier skill scores <0.2 as modest, 0.2–0.5 as moderate, 0.5–0.8 as substantial, and >0.8 as outstanding improvements in prediction.

To convert model probabilities into “yes/no” predictions, we selected 3 cutoffs for each antimicrobial: a balanced cutoff that optimally balances sensitivity and specificity, a high-sensitivity cutoff (sensitivity ≥95%), and a high-specificity cutoff (specificity ≥95%) [24]. These cutoffs were set using the training data and then applied unchanged to the test set. For each cutoff, we report sensitivity, specificity, positive and negative predictive values, and accuracy. We also report both overall and per-antimicrobial AUROC on the test set, interpreting AUROC values of 0.5–0.7 as poor, 0.7–0.8 good, 0.8–0.9 excellent, and >0.9 outstanding [25].

Given the rarity of nonsusceptible outcomes, particularly for carbapenems and antipseudomonal beta-lactam/BLI combinations, emphasis was placed on prevalence-aware metrics (AUPRC, positive predictive value [PPV], negative predictive value [NPV]) in addition to AUROC, in accordance with recommendations for imbalanced data [26].

Post hoc analyses to improve understanding and to conduct robustness checks of our findings are detailed in Supplementary Document 2. Sensitivity analyses were performed to assess the stability of the results under various clinically relevant assumptions. Additionally, subgroup analyses confirmed consistent model performance across some major patient and care-setting demographics.

### Ethics

The Institutional Review Board at the University of Iowa and the Research and Development Committee at the Iowa City Veterans Affairs Health Care System approved this study and granted a waiver of informed consent.

## RESULTS

### Description of the Cohort and Dataset

The developmental cohorts (training and validation) included 536 252 and 246 898 specimens from 287 045 and 139 377 unique patients for *E. coli* and *Klebsiella* spp., respectively. The test cohorts from 1 October 2023 to 30 September 2024 included 75 138 and 38 015 specimens from 53 913 and 26 907 unique patients, respectively. The descriptions of the cohorts were shown in Table 1 and Supplementary Table 2.

**Table 1.** Demographics of the Included Patients and Isolates

	*E. coli*		*Klebsiella* spp.	
	Training	Test	Training	Test
	(1/1/2017–9/30/2023)	(10/1/2023–9/30/2024)	(1/1/2017–9/30/2023)	(10/1/2023–9/30/2024)
Unique number of patients	287 045	53 913	139 377	26 907
Age (median/IQR)^a^	70 (61–77)	73 (63–78)	72 (65–78)	75 (67–79)
Female gender (%)^a^	69 117 (24.1%)	13 912 (25.8%)	16 422 (11.8%)	3163 (11.6%)
Rural residents (%)^a^	87 435 (30.5%)	15 848 (29.4%)	41 548 (29.8%)	7550 (30.0%)
Unique number of isolates	536 252	75 138	246 898	38 015
Specimen types				
Urine	472 620 (88.1%)	66 564 (88.6%)	200 322 (81.1%)	31 171 (82.0%)
Blood	20 856 (3.9%)	3090 (4.1%)	11 255 (4.6%)	1749 (4.6%)
Lower respiratory tract^b^	884 (0.2%)	110 (0.2%)	1229 (0.5%)	178 (0.5%)
Fecal/rectal swab^b^	3452 (0.6%)	313 (0.4%)	302 (0.1%)	22 (0.1%)
Other^b^	38 440 (7.2%)	5061 (6.7%)	33 790 (13.7%)	4895 (12.9%)
Prior microbiology data available	289 291 (54.0%)	43 496 (57.9%)	118 964 (48.2%)	19 624 (51.6%)
Prior antimicrobial exposures	422 780 (78.8%)	62 562 (83.3%)	197 144 (79.9%)	32 674 (86.0%)

^a^When 1 patient had more than 1 specimen during the dataset periods, the first appearance was used to describe the demographics.

^b^Lower respiratory tract, fecal/rectal swab, and other specimen types were combined as a single category for the model development purpose.

### Model Prediction Performance

For *E. coli*, the model achieved good overall performance (across all targets) with an AUROC of 0.786 on the training dataset (average cross-validation). On the test dataset, the model achieved a similarly good overall AUROC of 0.779 (Table 2). For *Klebsiella* spp., the model achieved an excellent performance with an overall AUROC of 0.817 on the training dataset (average cross-validation). On the test dataset, the model also achieved an excellent overall performance with an AUROC of 0.810 (Table 2).

**Table 2.** Per-Antimicrobial Model Performance on Training and Test Sets

Organism	Antimicrobial	Dataset	Prevalence of Resistance (%)	AUROC	Model Performance
*E. coli*	Aminopenicillins	Train	49.0%	0.749	Good
		Test	47.9%	0.753	Good
	NS cephalosporins	Train	26.9%	0.813	Excellent
		Test	26.2%	0.786	Good
	TMP/SMX	Train	25.3%	0.760	Good
		Test	24.1%	0.765	Good
	Fluoroquinolones	Train	33.5%	0.832	Excellent
		Test	31.0%	0.818	Excellent
	Aminopenicillin/BLI Combinations	Train	37.9%	0.750	Good
		Test	36.3%	0.743	Good
	ES cephalosporins	Train	11.2%	0.826	Excellent
		Test	12.9%	0.825	Excellent
	Antipseudomonal/BLI Combinations	Train	4.0%	0.770	Good
		Test	4.0%	0.762	Good
	Carbapenems	Train	0.2%	0.789	Good
		Test	0.3%	0.783	Good
*Klebsiella* spp.	NS cephalosporins	Train	26.2%	0.794	Good
		Test	26.8%	0.781	Good
	TMP/SMX	Train	13.4%	0.817	Excellent
		Test	15.2%	0.821	Excellent
	Fluoroquinolones	Train	13.5%	0.831	Excellent
		Test	16.6%	0.812	Excellent
	Aminopenicillin/BLI Combinations	Train	24.0%	0.750	Good
		Test	24.8%	0.763	Good
	ES cephalosporins	Train	10.3%	0.849	Excellent
		Test	12.8%	0.844	Excellent
	Antipseudomonal/BLI Combinations	Train	6.7%	0.811	Excellent
		Test	6.9%	0.800	Good
	Carbapenems	Train	1.4%	0.869	Excellent
		Test	1.4%	0.847	Excellent

Abbreviations: AUROC, area under the receiver operating characteristic curve; BLI, beta-lactamase inhibitor; ES, extended-spectrum; NS, narrow-spectrum; TMP/SMX, trimethoprim/sulfamethoxazole.

**Figure 1.**
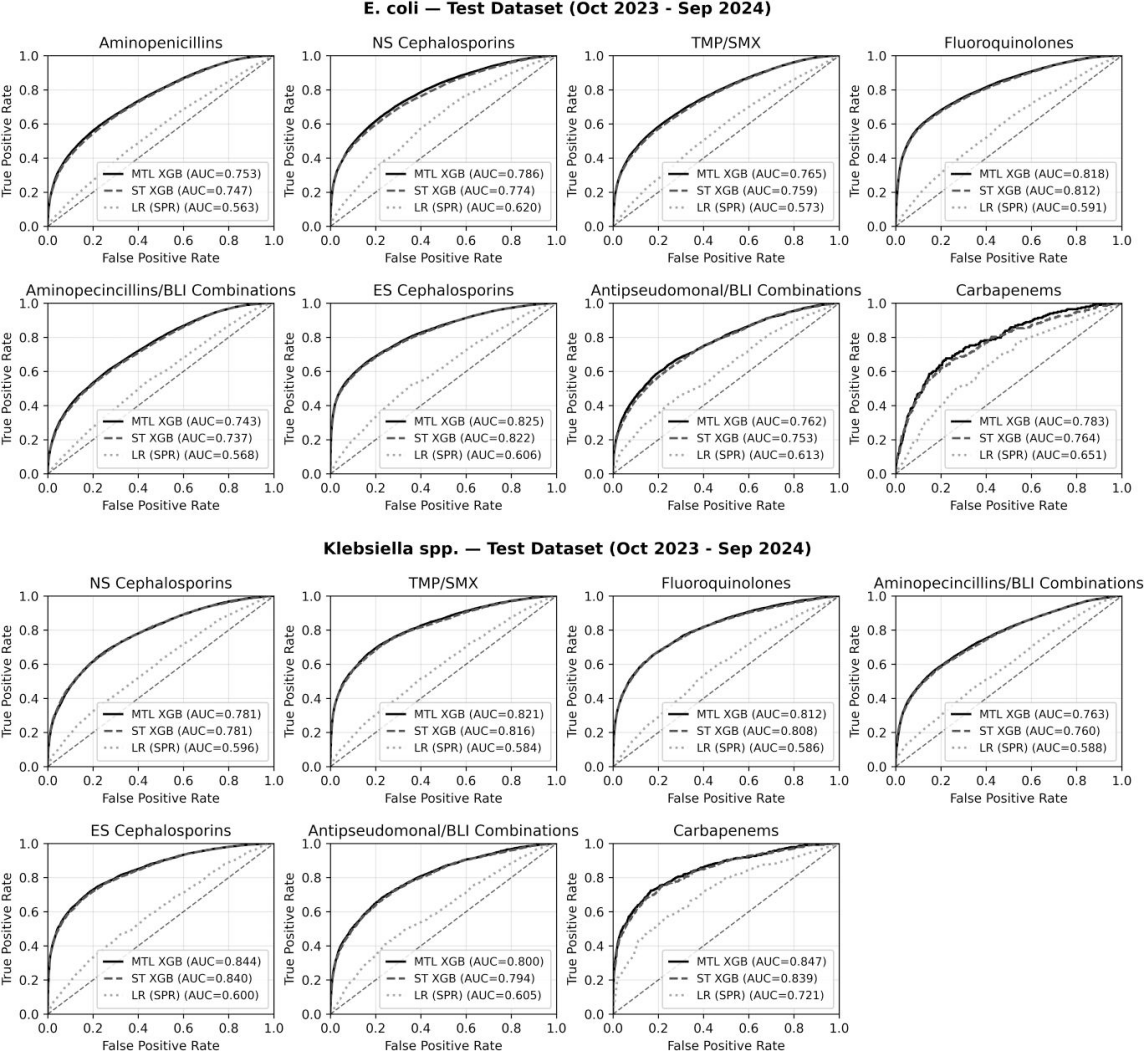
Per-antimicrobial ROC curves for *E. coli* and *Klebsiella* spp. Comparing multitask XGBoost, single-task XGBoost, and hospital antibiograms (logistic regression) on the test dataset. Abbreviations: AUC, area under the curve for receiver operating characteristic curve; LR(SPR), logistic regression based on hospital antibiograms; MTL XGB, multitask XGBoost; ROC, receiver operating characteristic; ST XGB, single-task XGBoost.

When evaluated for each antimicrobial class, the model achieved good to excellent performance across all prediction targets, with the highest AUROC observed for ES cephalosporins for *E. coli* (0.825) and carbapenems for *Klebsiella* spp. (0.847) and the lowest for aminopenicillin/BLI combinations for both *E. coli* (0.743) and *Klebsiella* spp. (0.763) (Table 2). Receiver operating characteristic curves for all included antimicrobial classes are shown in Figure 1.

Table 3 shows the diagnostic performances of models with 3 thresholds (balanced, sensitivity-prioritized, and specificity-prioritized). Balanced thresholds to maximize overall model performance generally yielded relatively low sensitivities (∼55%–70% in most cases) and higher specificities (∼75%–85% in most cases). When thresholds are prioritized to achieve sensitivities ≥95%, specificities decreased to ∼10%–30% in most cases, while thresholds to prioritize specificities (≥95%) decreased sensitivities to 20%–50%. More detailed PRCs for these 3 operating points (balanced, sensitivity ≥95%, and specificity ≥ 95%) are provided in Supplementary Document 1.

**Table 3.** Diagnostic Accuracy of Models With Three Sets of Thresholds for Predicted Probabilities

*E. coli*	Dataset	Prioritizing Overall Model Performance^a^			Prioritizing Sensitivities^b^			Prioritizing Specificities^c^		
		Th	Se	Sp	Th	Se	Sp	Th	Se	Sp
Aminopenicillins	Train	0.481	0.581	0.780	0.291	0.950	0.228	0.742	0.321	0.950
	Test	0.481	0.567	0.794	0.291	0.948	0.243	0.742	0.291	0.963
NS cephalosporins	Train	0.262	0.668	0.805	0.097	0.950	0.281	0.503	0.426	0.950
	Test	0.262	0.623	0.794	0.097	0.951	0.245	0.503	0.361	0.958
TMP/SMX	Train	0.238	0.580	0.806	0.115	0.950	0.211	0.468	0.360	0.950
	Test	0.238	0.565	0.816	0.115	0.953	0.224	0.468	0.333	0.958
Fluoroquinolones	Train	0.310	0.675	0.845	0.132	0.950	0.297	0.616	0.499	0.950
	Test	0.310	0.625	0.859	0.132	0.94	0.315	0.616	0.445	0.960
Aminopenicillin/BLI Combinations	Train	0.373	0.603	0.747	0.195	0.950	0.257	0.638	0.297	0.950
	Test	0.373	0.548	0.786	0.195	0.953	0.241	0.638	0.262	0.963
ES cephalosporins	Train	0.099	0.665	0.833	0.034	0.950	0.284	0.200	0.495	0.950
	Test	0.099	0.685	0.803	0.034	0.963	0.244	0.200	0.513	0.944
Antipseudomonal/BLI Combinations	Train	0.035	0.617	0.777	0.014	0.950	0.239	0.081	0.350	0.950
	Test	0.035	0.586	0.807	0.014	0.933	0.250	0.081	0.333	0.955
Carbapenems	Train	0.002	0.744	0.695	0.001	0.951	0.285	0.006	0.342	0.951
	Test	0.002	0.783	0.587	0.001	0.970	0.191	0.006	0.389	0.927
*Klebsiella* spp.	Dataset	Th	Se	Sp	Th	Se	Sp	Th	Se	Sp
NS cephalosporins	Train	0.255	0.653	0.781	0.100	0.950	0.271	0.460	0.388	0.950
	Test	0.255	0.681	0.738	0.100	0.965	0.204	0.460	0.388	0.936
TMP/SMX	Train	0.115	0.663	0.826	0.043	0.950	0.265	0.273	0.461	0.950
	Test	0.115	0.694	0.800	0.043	0.973	0.192	0.273	0.476	0.948
Fluoroquinolones	Train	0.113	0.694	0.818	0.043	0.950	0.300	0.281	0.475	0.950
	Test	0.113	0.694	0.776	0.043	0.972	0.174	0.281	0.465	0.942
Aminopenicillin/BLI Combinations	Train	0.224	0.595	0.770	0.100	0.950	0.202	0.439	0.337	0.950
	Test	0.224	0.635	0.752	0.100	0.965	0.168	0.439	0.377	0.948
ES cephalosporins	Train	0.082	0.713	0.833	0.029	0.950	0.322	0.199	0.530	0.950
	Test	0.082	0.734	0.793	0.029	0.977	0.217	0.199	0.537	0.941
Antipseudomonal/BLI Combinations	Train	0.058	0.661	0.819	0.020	0.950	0.258	0.150	0.441	0.950
	Test	0.058	0.659	0.791	0.020	0.968	0.194	0.150	0.417	0.947
Carbapenems	Train	0.011	0.728	0.857	0.003	0.950	0.369	0.025	0.588	0.950
	Test	0.011	0.709	0.842	0.003	0.950	0.310	0.025	0.524	0.945

Abbreviations: BLI, beta-lactamase inhibitor; ES, extended-spectrum; NS, narrow-spectrum; Se, sensitivity; Sp, specificity; Th, threshold; TMP/SMX, trimethoprim/sulfamethoxazole.

^a^Based on thresholds determined by the Youden index with training datasets.

^b^Based on thresholds to achieve sensitivities of at least 95% determined by the Youden index with training datasets.

^c^Based on thresholds to achieve specificities of at least 95% determined by the Youden index with training datasets.

### Comparison With Hospital Antibiogram and Conventional Single-task Extreme Gradient Boosting

Detailed comparison of the performance metrics is presented in Table 4 and Supplementary Table 3. Both single-task and multitask models demonstrated improvements in probabilistic prediction accuracies compared to conventional hospital antibiograms, especially for substantial to outstanding improvements for ES cephalosporins, antipseudomonal penicillins/BLI combinations, and carbapenems. Improvements were very similar between single-task and multitask models; however, false-negative rates for carbapenem resistance were lower in the multitask models, suggesting better calibration for isolates with true carbapenem resistance [27].

**Table 4.** Comparison With Hospital Antibiograms and Conventional Model

Metric		Conventional Hospital Antibiograms	Single-Target XGBoost Models	Multitask XGBoost Model
Individualized predictions		No	Yes	Yes
Simultaneous predictions		No	No	Yes
*E. coli* (on test dataset)				
Overall (combined) AUROC		N/A	N/A	0.862
Brier skill scores with interpretation for model improvements	Aminopenicillins	(Reference)	0.187 (modest improvement)	0.196 (modest improvement)
	NS cephalosporins	(Reference)	0.359 (moderate improvement)	0.373 (moderate improvement)
	TMP/SMX	(Reference)	0.395 (moderate improvement)	0.401 (substantial improvement)
	Fluoroquinolones	(Reference)	0.371 (moderate improvement)	0.379 (moderate improvement)
	Aminopenicillin/BLI combinations	(Reference)	0.208 (moderate improvement)	0.216 (moderate improvement)
	ES cephalosporins	(Reference)	0.699 (substantial improvement)	0.701 (outstanding improvement)
	Antipseudomonal/BLI combinations	(Reference)	0.845 (outstanding improvement)	0.847 (outstanding improvement)
	Carbapenems	(Reference)	0.989 (outstanding improvement)	0.989 (outstanding improvement)
False-negative rate for carbapenem Resistance^a^		39.90%	28.80%	21.70%
*Klebsiella* spp. (on test dataset)				
Overall (combined) AUROC		N/A	N/A	0.848
Brier skill scores with interpretation for model improvements	NS cephalosporins	(Reference)	0.398 (moderate improvement)	0.395 (moderate improvement)
	TMP/SMX	(Reference)	0.629 (substantial improvement)	0.630 (substantial improvement)
	Fluoroquinolones	(Reference)	0.607 (substantial improvement)	0.608 (substantial improvement)
	Aminopenicillin/BLI combinations	(Reference)	0.402 (substantial improvement)	0.404 (substantial improvement)
	ES cephalosporins	(Reference)	0.705 (outstanding improvement)	0.705 (outstanding improvement)
	Antipseudomonal/BLI combinations	(Reference)	0.771 (outstanding improvement)	0.773 (outstanding improvement)
	Carbapenems	(Reference)	0.933 (outstanding improvement)	0.934 (outstanding improvement)
False-negative rate for carbapenem resistance^a^		48.20%	28.50%	29.10%

Abbreviations: AUROC, area under the curve for receiver operating characteristic curve; BLI, beta-lactamase inhibitor; ES, extended-spectrum; NS, narrow-spectrum; TMP/SMX, trimethoprim/sulfamethoxazole.

^a^Based on thresholds determined by the Youden index with training datasets.

In post hoc gain-based feature-importance analyses, the multitask XGBoost model was primarily driven by the modeled antimicrobial target, prior susceptibility history, and recent antimicrobial exposures within clinically meaningful time windows (Supplementary Document 2). Antimicrobial-specific models showed the same overall pattern: each antimicrobial was most strongly influenced by its corresponding prior susceptibility result and by recent exposure to the same or related classes, with additional contributions from patient factors such as comorbidities and demographics (Supplementary Document 2).

## DISCUSSION

Our study aimed to develop and validate novel multitask XGBoost models to predict antimicrobial resistance profiles, accounting for mechanistic or epidemiological correlations among resistances across different antimicrobial classes in *E. coli* and *Klebsiella* spp. using structured EHR data. The model demonstrated robust overall performance, achieving overall AUROCs of around 0.8 for both *E. coli* and *Klebsiella* spp., as well as antimicrobial class-specific performances in the good to excellent range for all included targets. The model's ability to predict resistance to multiple antimicrobials simultaneously and create individualized predictions, including a list of resistance probabilities for each isolate (“personalized antibiogram”), highlights its potential utility in clinical settings. This capability is particularly notable for enhancing predictions and reducing false-negative rates for less prevalent but clinically significant carbapenem resistance. Detailed performance metrics for each antimicrobial were consistent across both the training and test datasets.

Compared to conventional hospital antibiograms, both the multitask and single-task XGBoost models demonstrated superior prediction performance, as expected. Single-target machine learning models, which incorporate extensive patient-level information and achieve substantially improved prediction accuracy compared with conventional antibiograms, often struggle with model training and poor calibration when predicting rare outcomes, such as carbapenem resistance [28–30]. In contrast, the multitask XGBoost model effectively leveraged the interdependence among resistance phenotypes, improving prediction accuracy and maintaining good performance even in low-endemicity contexts (carbapenem resistance). These findings align with previous studies that have highlighted the limitations of traditional methods and the potential of machine learning approaches to enhance antimicrobial resistance prediction [9, 31, 32].

The future implementation of this multitask XGBoost model in clinical practice offers several potential benefits. By providing accurate and individualized predictions of antimicrobial resistance, the model can enhance decision-making for empiric antimicrobial therapy. We view this as a decision-support adjunct to syndrome-based empiric protocols; prospective studies are needed to evaluate the impact on prescribing and outcomes.

Our study also demonstrated that dynamically setting specific thresholds for each target in this multitask framework, based on the intended purpose, can maximize clinical utility. For example, early detection of potential highly resistant or multidrug-resistant organisms with high sensitivity (eg, ≥95%) is ideal for empiric therapy selection for critically ill patients or infection control purposes because the negative prediction can reliably rule out resistance. Relatively low specificities or positive predictive values (eg, 15%–20%) can be tolerated in this context. Of note, our previous study demonstrated that hospital antibiograms had specificities at nearly 0% when interpretation thresholds were set to achieve sensitivities of ≥95% [1]. On the other hand, early detection of these organisms with high specificity (eg, ≥95%) can potentially facilitate the efficient enrollment of relevant patients in clinical trials evaluating the comparative effectiveness of novel agents for resistant organisms. For example, in the phase 3 trial that assessed the effectiveness of meropenem/vaborbactam compared to piperacillin/tazobactam in complicated urinary tract infection (TANGO I), there were only 3 patients who had meropenem-resistant isolates among over 500 participants, making it difficult to assess the specific effectiveness of this agent for most desired population (eg, patients with class A carbapenemase–producing Enterobacteriaceae infection) [33]. A multitask machine learning model, such as ours, can potentially serve as an identification tool by setting thresholds to prioritize specificities and increase the efficiency of including patients who likely benefit.

This study has several limitations. The model's reliance on historical data means that its predictions are only as accurate as the data on which it is trained. Continuous updating and validation of the model are essential to maintain its accuracy and relevance. We did not include resistance gene markers because testing was not consistently available across VA sites and years, particularly at smaller/rural facilities, and could introduce selection bias. Moreover, the study focused on *E. coli* and *Klebsiella* spp., and the findings may not be generalizable to other pathogens. Future research should explore the model's applicability to a broader range of organisms and antimicrobial agents. Lastly, the VHA population is generally older and overrepresented by males compared with the general US population, although ∼20% of isolates in this study originated from female patients, and the model had ample data points to learn from them. However, the VHA is the only integrated healthcare system in the United States that has a presence in all states, with varying prevalence rates of antimicrobial resistance, environmental factors, and all levels of care complexity. Therefore, it still provides an excellent opportunity to develop models that can adapt to various settings.

In summary, this study developed and validated a multitask XGBoost model to predict antimicrobial resistance profiles for common Enterobacteriaceae pathogens, *E. coli* and *Klebsiella* spp., using structured EHR data. The model demonstrated robust performance, outperforming conventional hospital antibiograms and single-target XGBoost classifiers. Its ability to generate personalized antibiograms offers significant improvements over traditional methods, enabling more precise and effective antimicrobial stewardship. While the study has limitations, its findings underscore the potential of machine learning approaches to enhance antimicrobial resistance prediction and support data-driven clinical decision-making.

## Supplementary Material

ciag027_Supplementary_Data

## Supplementary Data

Supplementary materials are available at *Clinical Infectious Diseases* online. Consisting of data provided by the authors to benefit the reader, the posted materials are not copyedited and are the sole responsibility of the authors, so questions or comments should be addressed to the corresponding author.

## References

[1] Hasegawa S, Livorsi DJ, Perencevich EN, Church JN, Goto M. Diagnostic accuracy of hospital antibiograms in predicting the risk of antimicrobial resistance in Enterobacteriaceae isolates: a nationwide multicenter evaluation at the Veterans Health Administration. Clin Infect Dis 2023; 77:1492–500. 37658908 10.1093/cid/ciad467 PMC11487110

[2] Antimicrobial resistance threats in the United States, 2021-2022. Centers for Disease Control and Prevention. Available at: https://www.cdc.gov/antimicrobial-resistance/data-research/threats/update-2022.html

[3] Wolford H, McCarthy NL, Baggs J, et al Antimicrobial-resistant infections in hospitalized patients. JAMA Netw Open 2025; 8:e2462059. 40085086 10.1001/jamanetworkopen.2024.62059 PMC11909612

[4] Duffy N, Li R, Czaja CA, et al Trends in incidence of carbapenem-resistant Enterobacterales in 7 US sites, 2016─2020. Open Forum Infect Dis 2023; 10:ofad609. 38130598 10.1093/ofid/ofad609 PMC10734676

[5] Tartof SY, Kuntz JL, Chen LH, et al Development and assessment of risk scores for carbapenem and extensive beta-lactam resistance among adult hospitalized patients with *Pseudomonas aeruginosa* infection. JAMA Netw Open 2018; 1:e183927. 30646267 10.1001/jamanetworkopen.2018.3927 PMC6324445

[6] Timbrook TT, Fowler MJ. Predicting extended-spectrum beta-lactamase and carbapenem resistance in Enterobacteriaceae bacteremia: a diagnostic model systematic review and meta-analysis. Antibiotics 2023; 12:1452. 37760748 10.3390/antibiotics12091452 PMC10525851

[7] Aslan AT, Ezure Y, Harris PNA, Paterson DL. Scoping review of risk-scoring tools for early prediction of bloodstream infections caused by carbapenem-resistant Enterobacterales: do we really have a reliable risk-scoring tool? JAC Antimicrob Resist 2024; 6:dlae032. 38414813 10.1093/jacamr/dlae032 PMC10899000

[8] Giannella M, Freire M, Rinaldi M, et al Development of a risk prediction model for carbapenem-resistant Enterobacteriaceae infection after liver transplantation: a multinational cohort study. Clin Infect Dis 2021; 73:e955–66. 33564840 10.1093/cid/ciab109

[9] McGuire RJ, Yu SC, Payne PRO, et al A pragmatic machine learning model to predict carbapenem resistance. Antimicrob Agents Chemother 2021; 65:e0006321. 33972243 10.1128/AAC.00063-21 PMC8218615

[10] Fernández L, Hancock REW. Adaptive and mutational resistance: role of porins and efflux pumps in drug resistance. Clin Microbiol Rev 2012; 25:661–81. 23034325 10.1128/CMR.00043-12 PMC3485749

[11] Moghnieh R, Abdallah D, Jadayel M, et al Epidemiology, risk factors, and prediction score of carbapenem resistance among inpatients colonized or infected with 3rd generation cephalosporin resistant Enterobacterales. Sci Rep 2021; 11:14757. 34285312 10.1038/s41598-021-94295-1 PMC8292374

[12] Critchley IA, Cotroneo N, Pucci MJ, Mendes R. The burden of antimicrobial resistance among urinary tract isolates of Escherichia coli in the United States in 2017. PLoS One 2019; 14:e0220265. 31821338 10.1371/journal.pone.0220265 PMC6903708

[13] Chen T . XGBoost: a scalable tree boosting system. Cornell University. **2016**

[14] Caruana R . Multitask learning. Machine learning 1997; 28:41–75.

[15] Diekema DJ, Hsueh PR, Mendes RE, et al The microbiology of bloodstream infection: 20-year trends from the SENTRY antimicrobial surveillance program. Antimicrob Agents Chemother 2019 63:e00355–19. 31010862 10.1128/AAC.00355-19 PMC6591610

[16] Holmes CL, Anderson MT, Mobley HLT, Bachman MA. Pathogenesis of gram-negative bacteremia. Clin Microbiol Rev 2021; 34:e00234–20. 33692149 10.1128/CMR.00234-20 PMC8549824

[17] Tindall BJ, Sutton G, Garrity GM. Enterobacter aerogenes Hormaeche and Edwards 1960 (approved lists 1980) and Klebsiella mobilis Bascomb et al. 1971 (approved lists 1980) share the same nomenclatural type (ATCC 13048) on the approved lists and are homotypic synonyms, with consequences for the name Klebsiella mobilis Bascomb et al. 1971 (approved lists 1980). Int J Syst Evol Microbiol 2017; 67:502–4. 27902205 10.1099/ijsem.0.001572

[18] Bhattacharjee D, Zhang T, Süsstrunk S. Salzmann M. Mult: An end-to-end multitask learning transformer **2022**:12031–41.

[19] VHA Directive 1106: pathology and laboratory medicine service. Veterans Health Administration. Available at: https://www.va.gov/vhapublications/publications.cfm. Accessed January 24, 2024.

[20] Report to congress: risk adjustment in Medicare advantage. Centers for Medicare & Medicaid Services. Available at: https://www.cms.gov/Medicare/Health-Plans/MedicareAdvtgSpecRateStats/Downloads/RTC-Dec2018.pdf

[21] Clinical classifications software for services and procedures. Agency for Healthcare Research and Quality. Available at: https://www.hcup-us.ahrq.gov/toolssoftware/ccs_svcsproc/ccssvcproc.jsp

[22] Collins GS, Moons KGM, Dhiman P, et al TRIPOD+AI statement: updated guidance for reporting clinical prediction models that use regression or machine learning methods. Bmj 2024; 385:e078378. 38626948 10.1136/bmj-2023-078378 PMC11019967

[23] Steyerberg EW, Vickers AJ, Cook NR, et al Assessing the performance of prediction models: a framework for traditional and novel measures. Research Support, Non-U.S. Gov't. Epidemiology 2010; 21:128–38. 20010215 10.1097/EDE.0b013e3181c30fb2 PMC3575184

[24] Perkins NJ, Schisterman EF. The inconsistency of “optimal” cutpoints obtained using two criteria based on the receiver operating characteristic curve. Am J Epidemiol 2006; 163:670–5. 16410346 10.1093/aje/kwj063 PMC1444894

[25] Hosmer DW Jr, Lemeshow S, Sturdivant RX. Applied logistic regression. vol 398. Wiley Series in Probability and Statistics. Hoboken, NJ: John Wiley & Sons, 2013.

[26] Saito T, Rehmsmeier M. The precision-recall plot is more informative than the ROC plot when evaluating binary classifiers on imbalanced datasets. PLoS One 2015; 10:e0118432. 25738806 10.1371/journal.pone.0118432 PMC4349800

[27] Hernández-Orallo J, Flach P, Ferri C. A unified view of performance metrics: translating threshold choice into expected classification loss. J Mach Learn Res 2012; 13:2813–69.

[28] Jiang Y, Wang HW, Tian FY, Guo Y, Wang XM. Predicting carbapenem-resistant Pseudomonas aeruginosa infection risk using XGBoost model and explainability. Sci Rep 2025; 15:19737. 40473759 10.1038/s41598-025-04028-x PMC12141430

[29] Zhang Q, Wu Y, Zhao L, et al Use of machine learning for real-time antibiotic treatment adjustment in high-risk patients with CRGNB infection. Comput Methods Programs Biomed 2025; 269:108847. 40570738 10.1016/j.cmpb.2025.108847

[30] He H, Garcia EA. Learning from imbalanced data. IEEE Transactions on knowledge and data engineering 2009; 21:1263–84.

[31] Goodman KE, Heil EL, Claeys KC, Banoub M, Bork JT. Real-world antimicrobial stewardship experience in a large academic medical center: using statistical and machine learning approaches to identify intervention “hotspots” in an antibiotic audit and feedback program. Open Forum Infect Dis 2022; 9:ofac289. 35873287 10.1093/ofid/ofac289 PMC9297307

[32] Beaudoin M, Kabanza F, Nault V, Valiquette L. Evaluation of a machine learning capability for a clinical decision support system to enhance antimicrobial stewardship programs. Artif Intell Med 2016; 68:29–36. 26947174 10.1016/j.artmed.2016.02.001

[33] Kaye KS, Bhowmick T, Metallidis S, et al Effect of meropenem-vaborbactam vs piperacillin-tazobactam on clinical cure or improvement and microbial eradication in complicated urinary tract infection: the TANGO I randomized clinical trial. JAMA 2018; 319:788–99. 29486041 10.1001/jama.2018.0438 PMC5838656

