# Supplementary material for: Personalized Antibiogram: A Novel Multitask Machine Learning Framework for Simultaneous Prediction of Antimicrobial Resistance Profile With Enhanced Detection of Carbapenem Resistance in Enterobacteriaceae
Source: Clin Infect Dis. 2026 Jan 17;83(1):e1–9. doi: 10.1093/cid/ciag027 (PMC13393128; doi:10.1093/cid/ciag027)
Supplement: ciag027_Supplementary_Data [file ciag027_supplementary_data.zip › Supplementary Document 1.docx]

**Supplementary Document 1: Model Performance Plots (balanced, high-sensitivity, and high-specificity PR curves)**

This supplementary document provides additional analyses to help interpret the model by including prevalence-aware and clinically interpretable metrics, specifically the area under the precision-recall curve (AUPRC), along with threshold-based sensitivity/specificity at clinically motivated operating points.

**
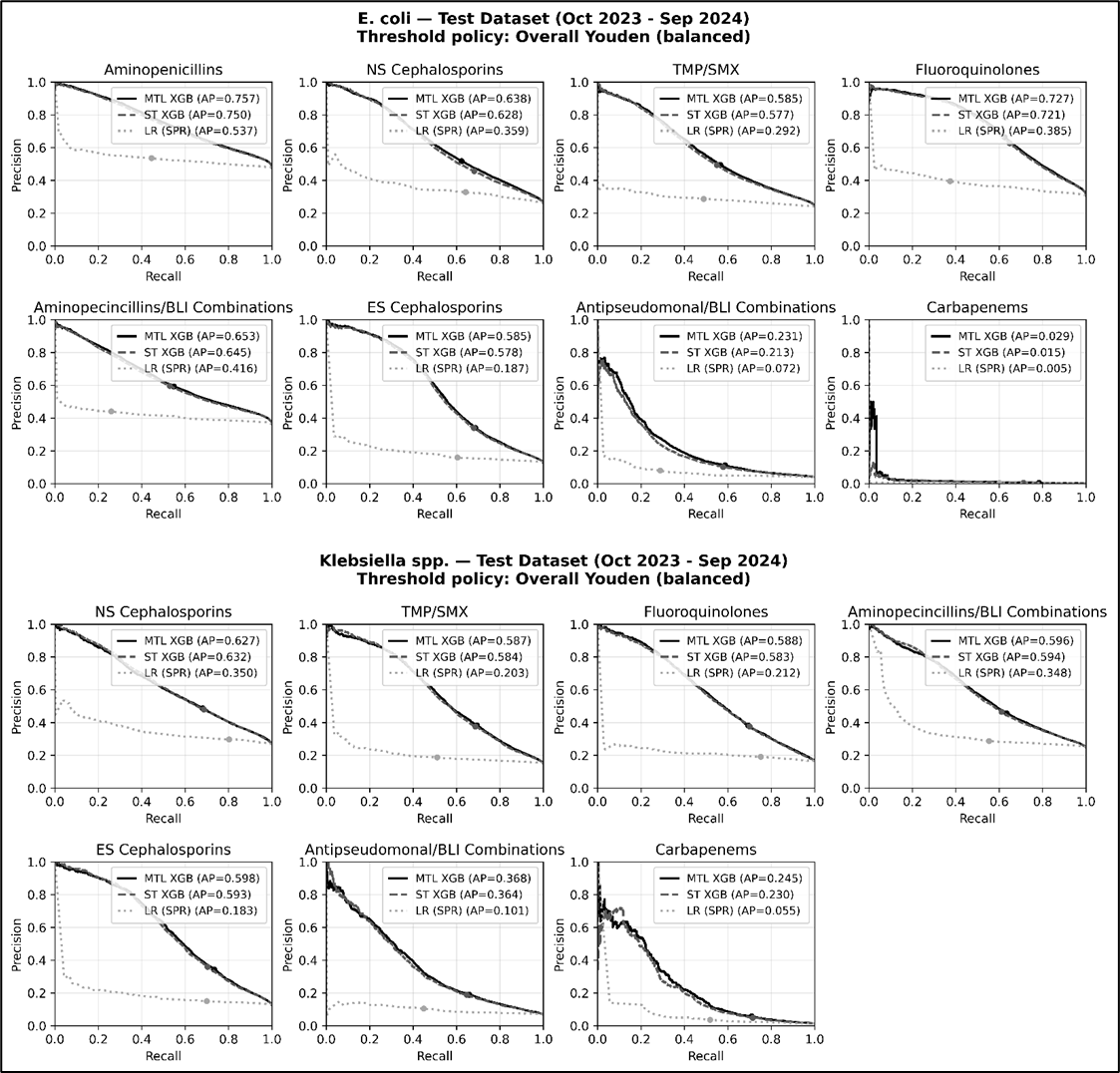
**

**Figure 1.** Precision-Recall Curves at the Balanced Operating Point (Youden-Optimal) on the test dataset.


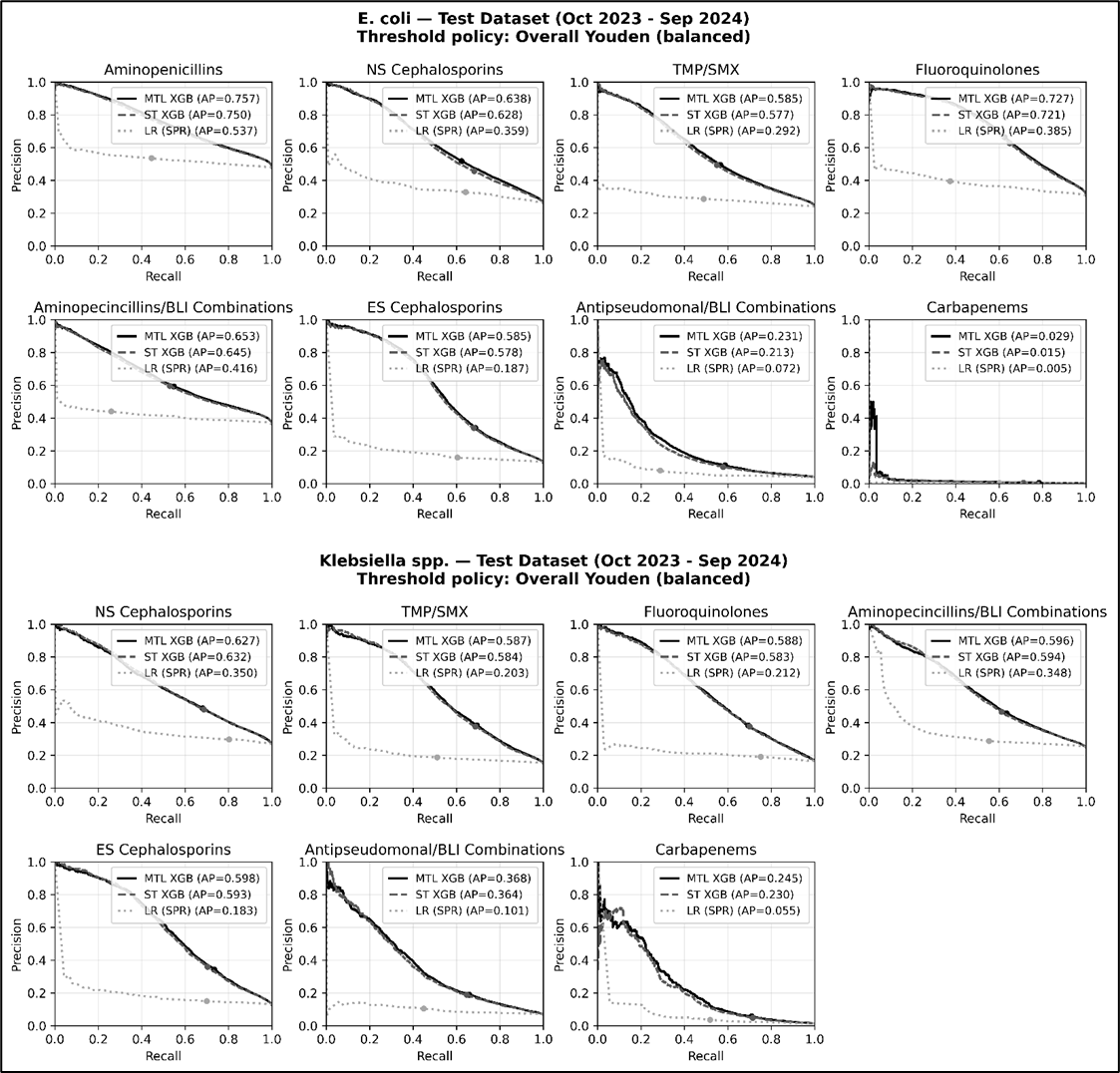


**Figure 2.** Precision-Recall Curves at a High-Sensitivity Operating Point (Sensitivity ≥ 0.95) on the test dataset.


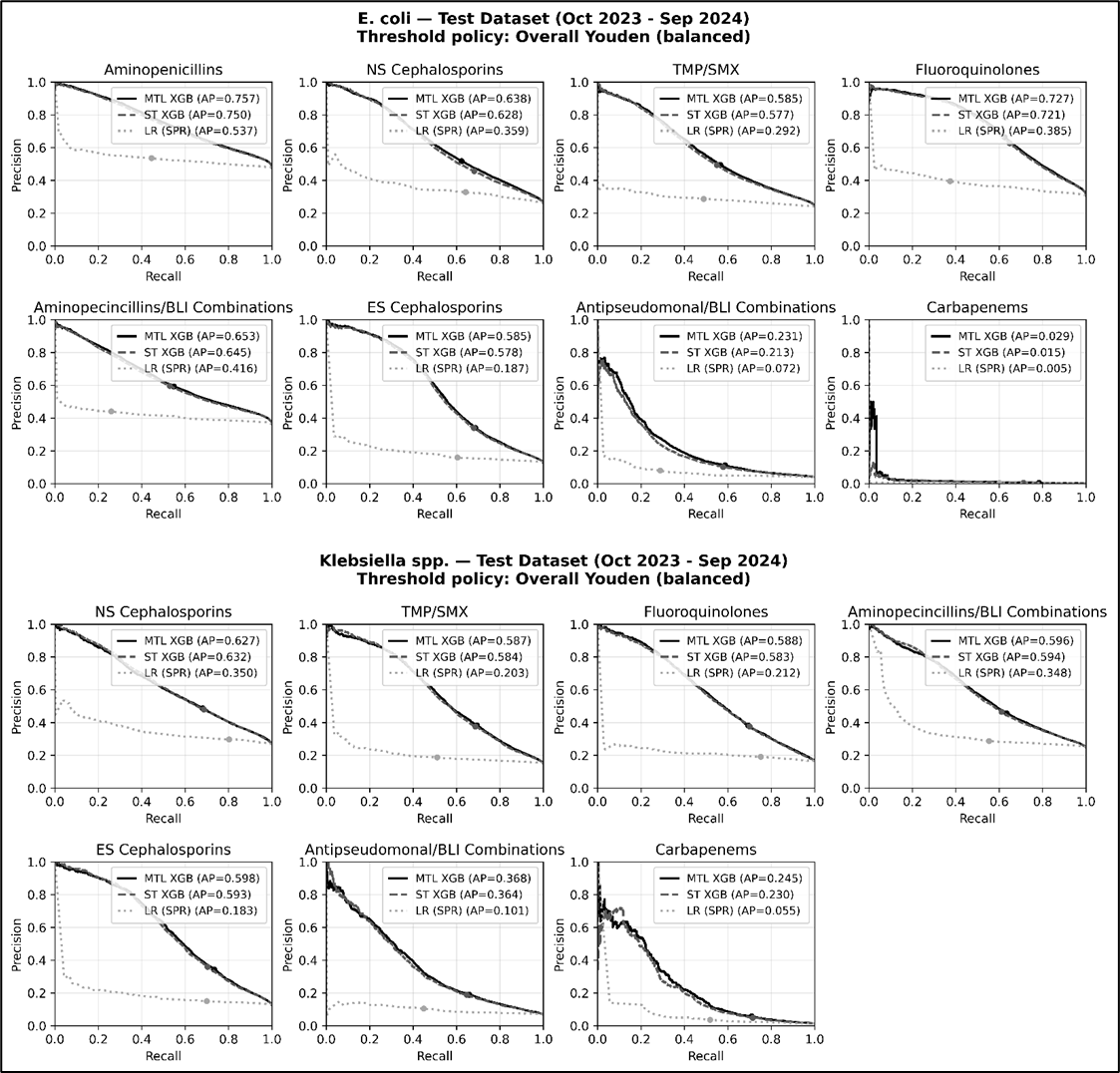


**Figure 3.** Precision-Recall Curves at a High-Specificity Operating Point (Specificity ≥ 0.95) on the test dataset.
