# Supplementary material for: Personalized Antibiogram: A Novel Multitask Machine Learning Framework for Simultaneous Prediction of Antimicrobial Resistance Profile With Enhanced Detection of Carbapenem Resistance in Enterobacteriaceae
Source: Clin Infect Dis. 2026 Jan 17;83(1):e1–9. doi: 10.1093/cid/ciag027 (PMC13393128; doi:10.1093/cid/ciag027)
Supplement: ciag027_Supplementary_Data [file ciag027_supplementary_data.zip › Supplementary Document 2.docx]

**Supplementary Document 2: Post-Hoc Sensitivity and Subgroup Analyses**

**Feature Importance**

We conducted a post hoc feature-importance analysis to identify which input variables most strongly contributed to the model’s predictions. We performed this after model development and did not modify the model's training labels, thresholds, or evaluation splits for this step. We focused on the XGBoost gain metric, which quantifies the improvement each feature contributed to the model during a tree split during training. Features with larger gain values contributed more to improving model fit and, therefore, were considered more influential in the model.

**Top features for the multi-task XGBoost model**

For the multi-task XGBoost model, we used the final fitted model trained on all antibiotics. We computed feature importance using XGBoost’s built-in gain-based importance function. We then created a ranked list of features by sorting the gain values from highest to lowest. To make the results easier to interpret, we also calculated the proportion of total gain for each feature by dividing each feature’s gain by the sum of gains across all features. We identified the highest-ranking features as the top drivers of the multi-task XGBoost model (**Table 1**).

**Top features for antibiotic-specific models**

To understand whether the most influential features differed by antibiotic, we repeated the gain-based importance analysis separately for each antibiotic outcome. For each antibiotic, we subset the training dataset to rows belonging to that antibiotic using the task identifier. We then trained a separate single-task XGBoost model on this antibiotic-specific subset, using the same model architecture, hyperparameters, and number of boosting iterations as the multi-task XGBoost model. This ensured that the feature-importance values were comparable across antibiotics by holding the learning settings constant. In these antibiotic-specific models, the task identifier was excluded as an input feature because it is constant within a single antibiotic subset and does not provide predictive information. After fitting each antibiotic-specific model, we extracted gain-based feature importance and ranked features within that antibiotic from highest to lowest gain (**Table 2**). We used these ranked lists to summarize the top features for each antibiotic model.

**How to interpret gain-based feature importance**

We focused on XGBoost’s gain-based feature importance, which summarizes the reduction in the model’s training loss attributable to splits on each feature; higher gain indicates a larger contribution to improving fit. It does not measure causality and does not indicate the direction of association; therefore, it does not directly indicate whether higher values of a feature increase or decrease the predicted probability of non-susceptibility. Gain can also be shared across correlated predictors, so strongly related clinical variables may divide importance between them. For these reasons, we interpreted gain as a practical summary of which variables the model relied on most for discrimination, rather than as evidence of causal effects.

**Table 1.** Top 20 features for the multi-task XGBoost model

| **Organism** | **Rank** | **Predictors** | **Gain (%)** | **Category** |
| --- | --- | --- | --- | --- |
| ***E. coli*** | 1 | Antibiotic target (modeled drug class) | 5.46 | - |
|  | 2 | Prior Aminopenicillins result | 1.28 | Prior susceptibility history |
|  | 3 | Prior Fluoroquinolones result | 0.96 | Prior susceptibility history |
|  | 4 | Prior ES Cephalosporins result | 0.66 | Prior susceptibility history |
|  | 5 | Fluoroquinolones use (31 to 90 days) | 0.62 | Recent antibiotic exposure |
|  | 6 | TMP/SMX use (4 to 7 days) | 0.60 | Recent antibiotic exposure |
|  | 7 | TMP/SMX use (8 to 14 days) | 0.60 | Recent antibiotic exposure |
|  | 8 | Carbapenems use (91 to 365 days) | 0.57 | Recent antibiotic exposure |
|  | 9 | Fluoroquinolones use (15 to 30 days) | 0.57 | Recent antibiotic exposure |
|  | 10 | Prior Aminopenicillins/BLI combinations result | 0.57 | Prior susceptibility history |
|  | 11 | Septicemia/Shock | 0.56 | Comorbidities (HCC) |
|  | 12 | Fluoroquinolones use (8 to 14 days) | 0.54 | Recent antibiotic exposure |
|  | 13 | Sex | 0.50 | Demographics |
|  | 14 | Prior TMP/SMX result | 0.48 | Prior susceptibility history |
|  | 15 | Fluoroquinolones use (4 to 7 days) | 0.47 | Recent antibiotic exposure |
|  | 16 | Diagnostic procedures, male genital | 0.47 | Procedures (CCS) |
|  | 17 | ES Cephalosporins use (8 to 14 days) | 0.47 | Recent antibiotic exposure |
|  | 18 | Fluoroquinolones use (1 to 3 days) | 0.45 | Recent antibiotic exposure |
|  | 19 | Aminopenicillins use (8 to 14 days) | 0.44 | Recent antibiotic exposure |
|  | 20 | TMP/SMX use (1 to 3 days) | 0.44 | Recent antibiotic exposure |
| ***Klebsiella* spp.** | 1 | Prior ES Cephalosporins result | 4.40 | Prior susceptibility history |
|  | 2 | Antibiotic target (modeled drug class) | 3.62 |  |
|  | 3 | TMP/SMX use (4 to 7 days) | 2.36 | Recent antibiotic exposure |
|  | 4 | Septicemia/Shock | 1.44 | Comorbidities (HCC) |
|  | 5 | Pressure of ulcer of skin with full thickness skin loss | 1.05 | Comorbidities (HCC) |
|  | 6 | Bone/Join/Muscle infections/necrosis | 0.98 | Comorbidities (HCC) |
|  | 7 | ES Cephalosporins use (4 to 7 days) | 0.91 | Recent antibiotic exposure |
|  | 8 | TMP/SMX use (8 to 14 days) | 0.90 | Recent antibiotic exposure |
|  | 9 | Fluoroquinolones use (4 to 7 days) | 0.87 | Recent antibiotic exposure |
|  | 10 | Prior Fluoroquinolones result | 0.86 | Prior susceptibility history |
|  | 11 | Fluoroquinolones use (8 to 14 days) | 0.84 | Recent antibiotic exposure |
|  | 12 | Aminopenicillins use (4 to 7 days) | 0.83 | Recent antibiotic exposure |
|  | 13 | Prior Fosfomycin result | 0.78 | Prior susceptibility history |
|  | 14 | Nitrofuran use (4 to 7 days) | 0.77 | Recent antibiotic exposure |
|  | 15 | TMP/SMX use (1 to 3 days) | 0.75 | Recent antibiotic exposure |
|  | 16 | Tetracycline use (4 to 7 days) | 0.69 | Recent antibiotic exposure |
|  | 17 | Prior TMP/SMX result | 0.65 | Prior susceptibility history |
|  | 18 | Aminopenicillins use (1 to 3 days) | 0.63 | Recent antibiotic exposure |
|  | 19 | Pressure ulcer of skin with necrosis through to muscle, tendon, or bone | 0.63 | Comorbidities (HCC) |
|  | 20 | ES Cephalosporins use (8 to 14 days) | 0.62 | Recent antibiotic exposure |

**Table 2.** Top 10 features for antibiotic-specific models

| Antibiotic | Rank | Predictors | Gain (%) | Category |
| --- | --- | --- | --- | --- |
| **E. coli** | | | | |
| Aminopenicillins | 1 | Prior Aminopenicillins result | 4.37 | Prior susceptibility history |
|  | 2 | Sex | 0.69 | Demographics |
|  | 3 | TMP/SMX use (4 to 7 days) | 0.68 | Recent antibiotic exposure |
|  | 4 | Fluoroquinolones use (15 to 30 days) | 0.61 | Recent antibiotic exposure |
|  | 5 | TMP/SMX use (8 to 14 days) | 0.59 | Recent antibiotic exposure |
|  | 6 | Aminopenicillins use (8 to 14 days) | 0.59 | Recent antibiotic exposure |
|  | 7 | Aminopenicillins use (4 to 7 days) | 0.55 | Recent antibiotic exposure |
|  | 8 | Carbapenems use (91 to 365 days) | 0.52 | Recent antibiotic exposure |
|  | 9 | Fluoroquinolones use (4 to 7 days) | 0.51 | Recent antibiotic exposure |
|  | 10 | Fluoroquinolones use (8 to 14 days) | 0.48 | Recent antibiotic exposure |
| NS Cephalosporins | 1 | Prior NS Cephalosporins result | 2.98 | Prior susceptibility history |
|  | 2 | Hospital antibiogram % for NS Cephalosporins | 0.84 | Facility-level baseline rates |
|  | 3 | Septicemia/Shock | 0.72 | Comorbidities (HCC) |
|  | 4 | Carbapenems use (91 to 365 days) | 0.68 | Recent antibiotic exposure |
|  | 5 | ES Cephalosporins use (8 to 14 days) | 0.65 | Recent antibiotic exposure |
|  | 6 | Sex | 0.61 | Demographics |
|  | 7 | Carbapenems use (31 to 90 days) | 0.59 | Recent antibiotic exposure |
|  | 8 | Aminopenicillins use (4 to 7 days) | 0.52 | Recent antibiotic exposure |
|  | 9 | ES Cephalosporins use (1 to 3 days) | 0.52 | Recent antibiotic exposure |
|  | 10 | Prior ES Cephalosporins result | 0.51 | Prior susceptibility history |
| TMP/SMX | 1 | Prior TMP/SMX result | 3.81 | Prior susceptibility history |
|  | 2 | TMP/SMX use (8 to 14 days) | 1.83 | Recent antibiotic exposure |
|  | 3 | TMP/SMX use (4 to 7 days) | 1.70 | Recent antibiotic exposure |
|  | 4 | TMP/SMX use (1 to 3 days) | 0.83 | Recent antibiotic exposure |
|  | 5 | TMP/SMX use (15 to 30 days) | 0.56 | Recent antibiotic exposure |
|  | 6 | Tetracycline use (15 to 30 days) | 0.52 | Recent antibiotic exposure |
|  | 7 | TMP/SMX use (31 to 90 days) | 0.51 | Recent antibiotic exposure |
|  | 8 | HIV/AIDS | 0.50 | Comorbidities (HCC) |
|  | 9 | Tetracycline use (8 to 14 days) | 0.47 | Recent antibiotic exposure |
|  | 10 | Fluoroquinolones use (15 to 30 days) | 0.46 | Recent antibiotic exposure |
| Fluoroquinolones | 1 | Prior Fluoroquinolones result | 5.80 | Prior susceptibility history |
|  | 2 | Fluoroquinolones use (31 to 90 days) | 2.13 | Recent antibiotic exposure |
|  | 3 | Fluoroquinolones use (8 to 14 days) | 1.64 | Recent antibiotic exposure |
|  | 4 | Fluoroquinolones use (15 to 30 days) | 1.11 | Recent antibiotic exposure |
|  | 5 | Sex | 1.04 | Demographics |
|  | 6 | Fluoroquinolones use (4 to 7 days) | 0.90 | Recent antibiotic exposure |
|  | 7 | Fluoroquinolones use (91 to 365 days) | 0.75 | Recent antibiotic exposure |
|  | 8 | Fluoroquinolones use (1 to 3 days) | 0.67 | Recent antibiotic exposure |
|  | 9 | Carbapenems use (91 to 365 days) | 0.59 | Recent antibiotic exposure |
|  | 10 | Diagnostic procedures, male genital | 0.55 | Procedures (CCS) |
| Aminopenicillins/  BLI Combinations | 1 | Prior Aminopenicillins/BLI combinations result | 3.44 | Prior susceptibility history |
|  | 2 | Aminopenicillins use (8 to 14 days) | 0.69 | Recent antibiotic exposure |
|  | 3 | Aminopenicillins use (4 to 7 days) | 0.60 | Recent antibiotic exposure |
|  | 4 | TMP/SMX use (8 to 14 days) | 0.54 | Recent antibiotic exposure |
|  | 5 | Sex | 0.51 | Demographics |
|  | 6 | Prior Aminopenicillins result | 0.51 | Prior susceptibility history |
|  | 7 | TMP/SMX use (4 to 7 days) | 0.50 | Recent antibiotic exposure |
|  | 8 | Aminopenicillins use (1 to 3 days) | 0.45 | Recent antibiotic exposure |
|  | 9 | Aminopenicillins/BLI combinations use (8 to 14 days) | 0.44 | Recent antibiotic exposure |
|  | 10 | Fluoroquinolones use (15 to 30 days) | 0.44 | Recent antibiotic exposure |
| ES Cephalosporins | 1 | Prior ES Cephalosporins result | 4.79 | Prior susceptibility history |
|  | 2 | Carbapenems use (91 to 365 days) | 1.71 | Recent antibiotic exposure |
|  | 3 | Septicemia/Shock | 1.45 | Comorbidities (HCC) |
|  | 4 | ES Cephalosporins use (8 to 14 days) | 0.94 | Recent antibiotic exposure |
|  | 5 | Carbapenems use (31 to 90 days) | 0.90 | Recent antibiotic exposure |
|  | 6 | Sex | 0.67 | Demographics |
|  | 7 | ES Cephalosporins use (1 to 3 days) | 0.64 | Recent antibiotic exposure |
|  | 8 | Routine chest X-ray | 0.64 | Procedures (CCS) |
|  | 9 | Carbapenems use (15 to 30 days) | 0.62 | Recent antibiotic exposure |
|  | 10 | Fosfomycin use (31 to 90 days) | 0.59 | Recent antibiotic exposure |
| Antipseudomonal/  BLI Combinations | 1 | Prior Antipseudomonal/BLI combinations result | 1.30 | Prior susceptibility history |
|  | 2 | Antipseudomonal/BLI combinations use (8 to 14 days) | 1.23 | Recent antibiotic exposure |
|  | 3 | Septicemia/Shock | 0.89 | Comorbidities (HCC) |
|  | 4 | Antipseudomonal/BLI combinations use (4 to 7 days) | 0.83 | Recent antibiotic exposure |
|  | 5 | Antipseudomonal/BLI combinations use (15 to 30 days) | 0.67 | Recent antibiotic exposure |
|  | 6 | ES Cephalosporins/BLI combinations use (91 to 365 days) | 0.60 | Recent antibiotic exposure |
|  | 7 | Aminopenicillins use (4 to 7 days) | 0.59 | Recent antibiotic exposure |
|  | 8 | PCN use (8 to 14 days) | 0.56 | Recent antibiotic exposure |
|  | 9 | Hemodialysis | 0.54 | Procedures (CCS) |
|  | 10 | Aminopenicillins use (1 to 3 days) | 0.53 | Recent antibiotic exposure |
| Carbapenems | 1 | Carbapenems use (4 to 7 days) | 1.34 | Recent antibiotic exposure |
|  | 2 | Carbapenems use (8 to 14 days) | 0.91 | Recent antibiotic exposure |
|  | 3 | Carbapenems use (1 to 3 days) | 0.69 | Recent antibiotic exposure |
|  | 4 | Hemodialysis | 0.61 | Procedures (CCS) |
|  | 5 | Sex | 0.57 | Demographics |
|  | 6 | Prior ES Cephalosporins/BLI combinations result | 0.57 | Prior susceptibility history |
|  | 7 | Carbapenems use (15 to 30 days) | 0.57 | Recent antibiotic exposure |
|  | 8 | Septicemia/Shock | 0.56 | Comorbidities (HCC) |
|  | 9 | Chronic Kidney Disease, Stage 5 | 0.56 | Comorbidities (HCC) |
|  | 10 | Colorectal resection | 0.54 | Procedures (CCS) |
| **Klebsiella spp.** | | | | |
| NS Cephalosporins | 1 | Prior NS Cephalosporins result | 3.63 | Prior susceptibility history |
|  | 2 | ES Cephalosporins use (4 to 7 days) | 1.91 | Recent antibiotic exposure |
|  | 3 | Bone/Join/Muscle Infections/Necrosis | 1.34 | Comorbidities (HCC) |
|  | 4 | ES Cephalosporins use (8 to 14 days) | 1.19 | Recent antibiotic exposure |
|  | 5 | Aminopenicillins use (4 to 7 days) | 1.09 | Recent antibiotic exposure |
|  | 6 | Fluoroquinolones use (8 to 14 days) | 1.01 | Recent antibiotic exposure |
|  | 7 | Fluoroquinolones use (4 to 7 days) | 0.86 | Recent antibiotic exposure |
|  | 8 | Carbapenems use (15 to 30 days) | 0.84 | Recent antibiotic exposure |
|  | 9 | Carbapenems use (91 to 365 days) | 0.81 | Recent antibiotic exposure |
|  | 10 | Aminopenicillins use (1 to 3 days) | 0.76 | Recent antibiotic exposure |
| TMP/SMX | 1 | TMP/SMX use (4 to 7 days) | 8.80 | Recent antibiotic exposure |
|  | 2 | Prior TMP/SMX result | 4.88 | Prior susceptibility history |
|  | 3 | TMP/SMX use (8 to 14 days) | 2.31 | Recent antibiotic exposure |
|  | 4 | Pressure of Ulcer of Skin with Full Thickness Skin Loss | 1.70 | Comorbidities (HCC) |
|  | 5 | TMP/SMX use (1 to 3 days) | 1.43 | Recent antibiotic exposure |
|  | 6 | Fluoroquinolones use (4 to 7 days) | 1.27 | Recent antibiotic exposure |
|  | 7 | ES Cephalosporins use (4 to 7 days) | 1.24 | Recent antibiotic exposure |
|  | 8 | Prior Fosfomycin result | 0.96 | Prior susceptibility history |
|  | 9 | Septicemia/Shock | 0.91 | Comorbidities (HCC) |
|  | 10 | TMP/SMX use (15 to 30 days) | 0.91 | Recent antibiotic exposure |
| Fluoroquinolones | 1 | Prior Fluoroquinolones result | 5.16 | Prior susceptibility history |
|  | 2 | Fluoroquinolones use (4 to 7 days) | 1.89 | Recent antibiotic exposure |
|  | 3 | Nitrofuran use (4 to 7 days) | 1.66 | Recent antibiotic exposure |
|  | 4 | Septicemia/Shock | 1.48 | Comorbidities (HCC) |
|  | 5 | Pressure of Ulcer of Skin with Full Thickness Skin Loss | 1.45 | Comorbidities (HCC) |
|  | 6 | Fluoroquinolones use (8 to 14 days) | 1.42 | Recent antibiotic exposure |
|  | 7 | TMP/SMX use (4 to 7 days) | 1.19 | Recent antibiotic exposure |
|  | 8 | Nitrofuran use (1 to 3 days) | 1.14 | Recent antibiotic exposure |
|  | 9 | Pressure Ulcer of Skin with Necrosis Through to Muscle, Tendon, or Bone | 1.14 | Comorbidities (HCC) |
|  | 10 | ES Cephalosporins use (4 to 7 days) | 1.12 | Recent antibiotic exposure |
| Aminopenicillins/  BLI Combinations | 1 | Prior Aminopenicillins/BLI combinations result | 2.84 | Prior susceptibility history |
|  | 2 | Bone/Join/Muscle Infections/Necrosis | 1.50 | Comorbidities (HCC) |
|  | 3 | Aminopenicillins use (4 to 7 days) | 1.33 | Recent antibiotic exposure |
|  | 4 | ES Cephalosporins use (4 to 7 days) | 1.26 | Recent antibiotic exposure |
|  | 5 | Fluoroquinolones use (8 to 14 days) | 1.11 | Recent antibiotic exposure |
|  | 6 | Tetracycline use (8 to 14 days) | 1.10 | Recent antibiotic exposure |
|  | 7 | Tetracycline use (4 to 7 days) | 1.07 | Recent antibiotic exposure |
|  | 8 | Fluoroquinolones use (4 to 7 days) | 1.04 | Recent antibiotic exposure |
|  | 9 | Aminopenicillins use (1 to 3 days) | 1.01 | Recent antibiotic exposure |
|  | 10 | ES Cephalosporins use (8 to 14 days) | 0.99 | Recent antibiotic exposure |
| ES Cephalosporins | 1 | Prior ES Cephalosporins result | 7.65 | Prior susceptibility history |
|  | 2 | Septicemia/Shock | 3.85 | Comorbidities (HCC) |
|  | 3 | Pressure of Ulcer of Skin with Full Thickness Skin Loss | 1.50 | Comorbidities (HCC) |
|  | 4 | ES Cephalosporins use (4 to 7 days) | 1.34 | Recent antibiotic exposure |
|  | 5 | Fluoroquinolones use (4 to 7 days) | 1.10 | Recent antibiotic exposure |
|  | 6 | Carbapenems use (91 to 365 days) | 1.10 | Recent antibiotic exposure |
|  | 7 | Carbapenems use (15 to 30 days) | 1.06 | Recent antibiotic exposure |
|  | 8 | Prior Fosfomycin result | 1.04 | Prior susceptibility history |
|  | 9 | ES Cephalosporins use (8 to 14 days) | 0.95 | Recent antibiotic exposure |
|  | 10 | TMP/SMX use (4 to 7 days) | 0.76 | Recent antibiotic exposure |
| Antipseudomonal/  BLI Combinations | 1 | Prior Antipseudomonal/BLI combinations result | 3.04 | Prior susceptibility history |
|  | 2 | Septicemia/Shock | 2.80 | Comorbidities (HCC) |
|  | 3 | ES Cephalosporins use (4 to 7 days) | 0.93 | Recent antibiotic exposure |
|  | 4 | Respirator Dependence/Tracheostomy Status | 0.91 | Comorbidities (HCC) |
|  | 5 | Bone/Join/Muscle Infections/Necrosis | 0.91 | Comorbidities (HCC) |
|  | 6 | Fluoroquinolones use (4 to 7 days) | 0.90 | Recent antibiotic exposure |
|  | 7 | Aminopenicillins use (4 to 7 days) | 0.85 | Recent antibiotic exposure |
|  | 8 | ES Cephalosporins use (8 to 14 days) | 0.83 | Recent antibiotic exposure |
|  | 9 | Prior Aminopenicillins/BLI combinations result | 0.81 | Prior susceptibility history |
|  | 10 | Aminopenicillins use (1 to 3 days) | 0.78 | Recent antibiotic exposure |
| Carbapenems | 1 | Prior Carbapenems result | 3.52 | Prior susceptibility history |
|  | 2 | Respirator Dependence/Tracheostomy Status | 2.09 | Comorbidities (HCC) |
|  | 3 | Hospital antibiogram % for Carbapenems | 1.74 | Facility-level baseline rates |
|  | 4 | Bone/Join/Muscle Infections/Necrosis | 1.22 | Comorbidities (HCC) |
|  | 5 | ES Cephalosporins/BLI combinations use (31 to 90 days) | 1.00 | Recent antibiotic exposure |
|  | 6 | Prior Antipseudomonal/BLI combinations result | 0.87 | Prior susceptibility history |
|  | 7 | Carbapenems use (4 to 7 days) | 0.85 | Recent antibiotic exposure |
|  | 8 | Pressure Ulcer of Skin with Necrosis Through to Muscle, Tendon, or Bone | 0.81 | Comorbidities (HCC) |
|  | 9 | Carbapenems use (8 to 14 days) | 0.81 | Recent antibiotic exposure |
|  | 10 | Prior Polymyxin result | 0.77 | Prior susceptibility history |

**Sensitivity and Subgroup Analyses**

All sensitivity and subgroup analyses were conducted after the main model was trained. We did not retrain or refit any model for these analyses. We reused the same predicted probabilities and antibiotic-specific decision thresholds as in the main analysis. For the training period, we used out-of-fold predicted probabilities from cross-validation. For the test period, we used the held-out predicted probabilities. We recomputed all performance metrics after applying each sensitivity rule or subgroup filter.

We summarize performance using AUC and false negative rate (FNR). AUC reflects how well the model separates non-susceptible from susceptible outcomes across all probability thresholds, so higher values indicate better separation. FNR reflects the proportion of non-susceptible cases that the model misses at the fixed antibiotic-specific decision thresholds used in the main analysis; lower values indicate fewer missed non-susceptible cases. Any differences reflect changes in which observations were included in the evaluation rather than changes to the model itself.

**Sensitivity analysis S1: Removing Fluoroquinolones Outcomes Before 2019**

In 2019, the Clinical and Laboratory Standards Institute (CLSI) lowered Fluoroquinolones MIC breakpoints for Enterobacterales, which can reclassify some previously “susceptible” isolates as “non-susceptible.” To assess whether this change materially affected our results, we performed a sensitivity analysis excluding Fluoroquinolones outcomes before January 2019. Fluoroquinolones rows were identified using the task identifiers used in our pipeline.

When outcomes from Fluoroquinolones before 2019 were excluded, test set performance remained unchanged (**Figure 1**). Across all antibiotics and both organisms, AUC and FNR values were identical between the baseline and sensitivity columns. This indicates that older pre-2019 Fluoroquinolones observations do not drive the reported performance on the test set. In practice, this pattern is consistent with the test set containing few or no such older Fluoroquinolones rows, or with their exclusion being too small to affect the test summaries materially.


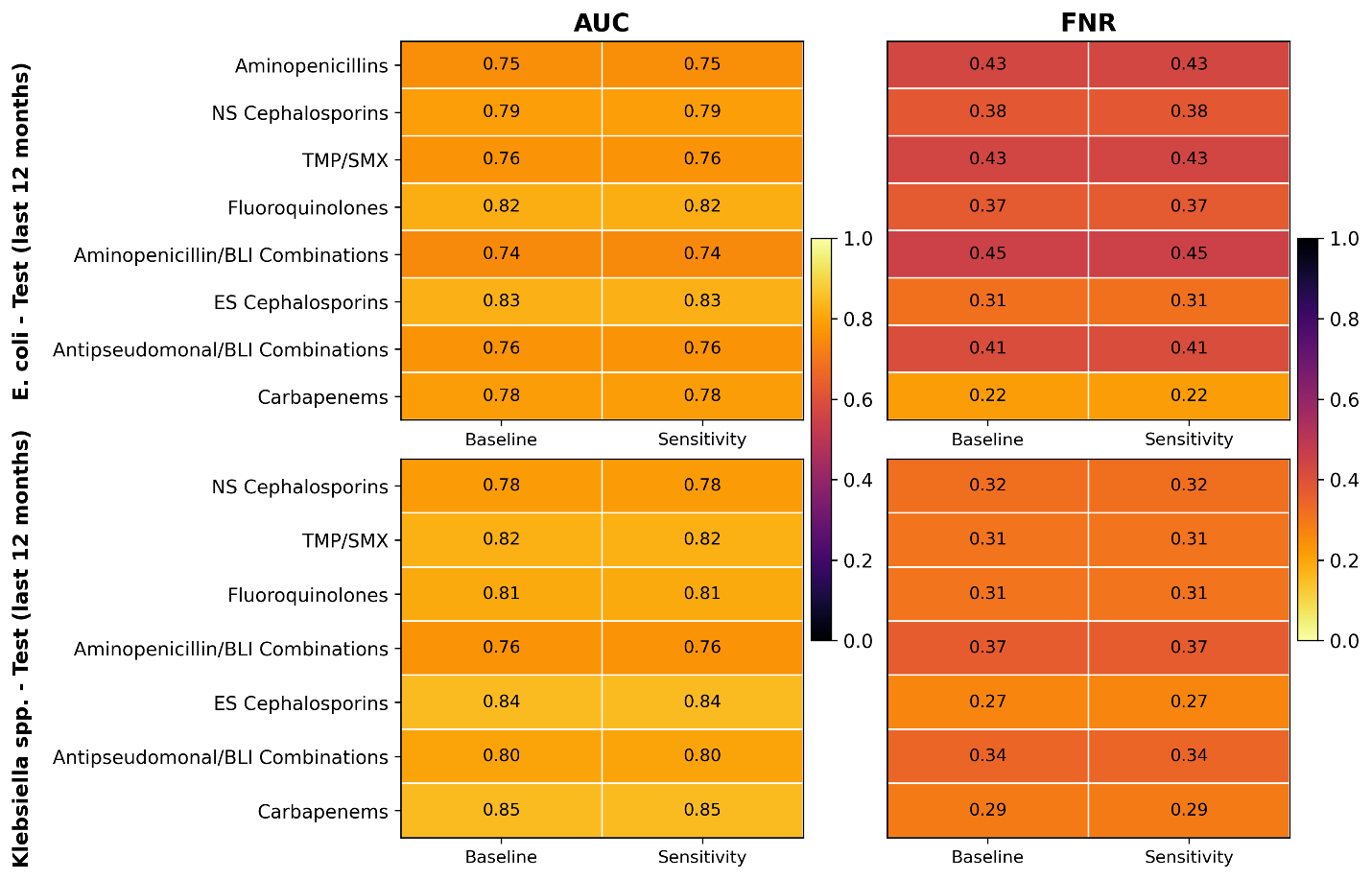


**Figure 1**. Heatmaps comparing test-set AUC (discrimination) and FNR for each antibiotic class and organism, between the baseline and sensitivity analyses, excluding fluoroquinolone outcomes before January 2019.

**Sensitivity analysis S2: First isolate per patient × organism per calendar year**

We performed a post hoc sensitivity analysis to reduce the influence of repeated isolates from the same patient within a year. This analysis required accurate specimen timestamps to enable ordering of isolates. We therefore loaded the original microbiology file that contains the specimen identifier and specimen collection time. We merged this specimen collection time into the long-format training and test tables using the specimen identifier. Rows with missing timestamps were retained because they could not be reliably ordered.

Restricting the evaluation to the first isolate per patient per year resulted in modestly lower performance for most antibiotics in both organisms. Across antibiotics, AUC was generally lower than the baseline values, typically by a few hundredths (**Figure 2**). At the same time, FNR was generally higher, often by several percentage points. This pattern suggests that removing repeated isolates within a patient makes the prediction task more difficult. Under this stricter definition, the model still performs in the same overall range. Still, it shows weaker separation and misses a larger fraction of non-susceptible cases at the fixed thresholds.


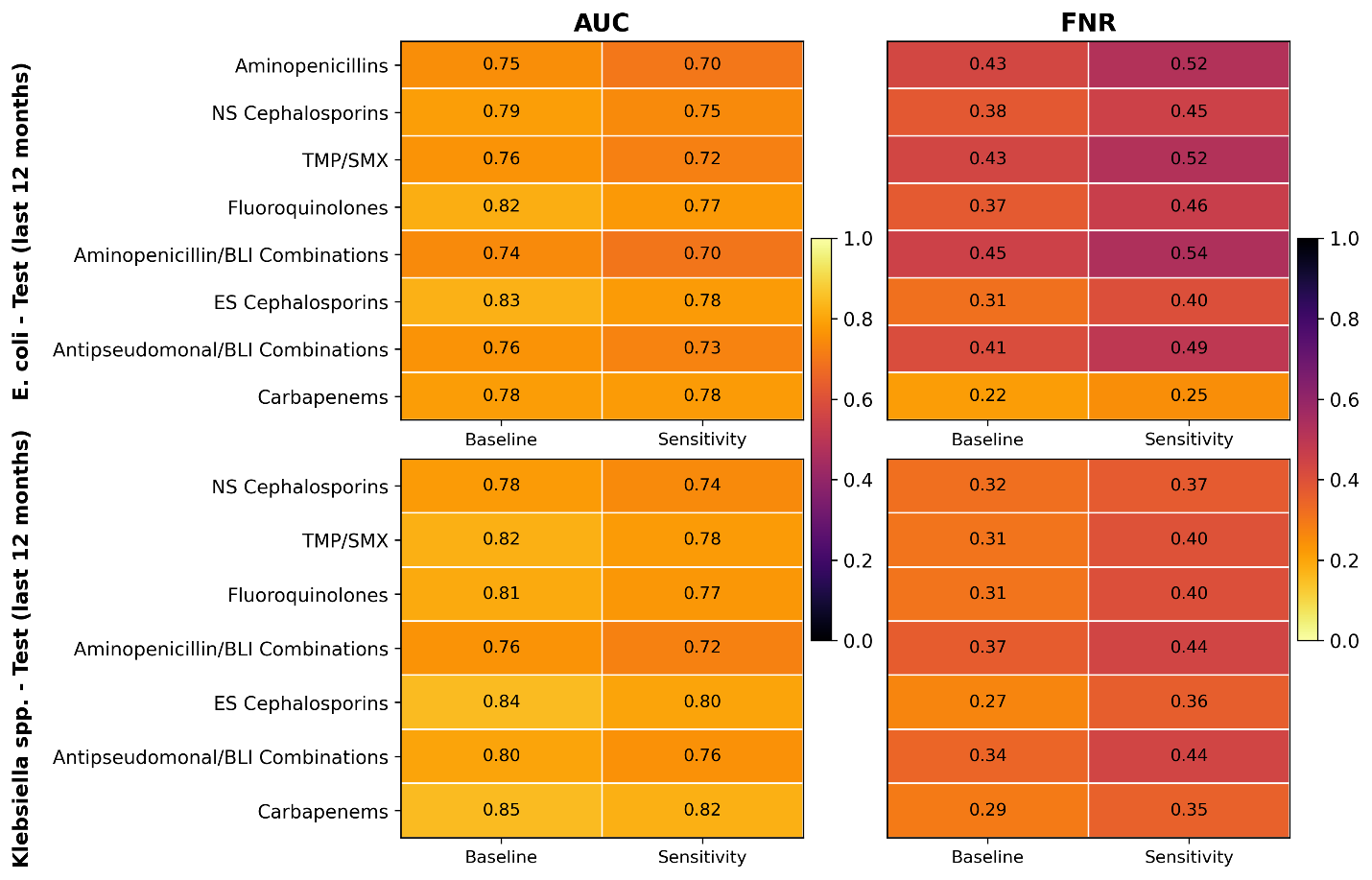


**Figure 2**. Heatmaps showing test-set AUC and FNR by antibiotic class for *E. coli* and *Klebsiella* spp. when the evaluation is restricted to the first isolate per patient, per organism, per calendar year, compared with the baseline analysis.

**Sensitivity analysis S3: Censor after the first non-susceptible event per patient and antibiotic**

We performed a post hoc sensitivity analysis to test whether later observations after a patient’s first non-susceptible result were driving performance. Using the merged specimen collection time, we identified for each patient and each antibiotic task the earliest time at which the label indicated non-susceptibility. We then censored the data by keeping only rows that occurred on or before that first non-susceptible time for that patient and task. This approach retains the first non-susceptible event and removes later follow-up rows that may be strongly correlated with it. Rows with missing timestamps were retained because censoring could not be applied reliably.

Censoring observations after the first non-susceptible event produced a pattern like the first isolate analysis. For most antibiotics and across both organisms, AUC decreased relative to baseline, whereas FNR increased (**Figure 3**). This indicates that performance in the baseline evaluation is partly supported by later observations that occur after resistance first appears for a patient and antibiotic, which may be easier for the model to identify because they follow a clear resistance signal in the patient’s history. After censoring, the model continues to perform comparably overall, but with a modest reduction in discrimination and a higher FNR at the same thresholds.


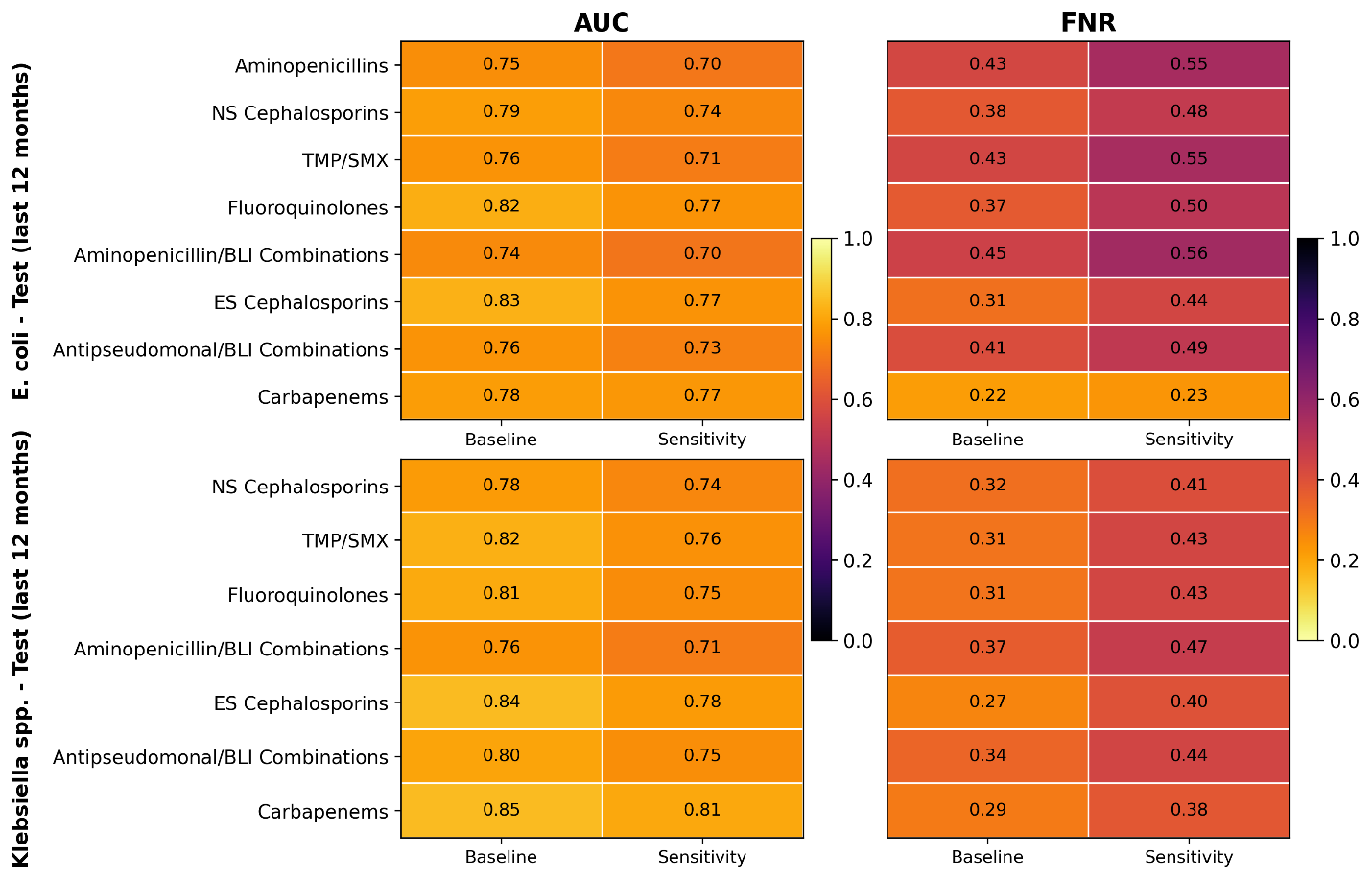


**Figure 3**. Heatmaps comparing baseline vs censoring after first non-susceptible evaluation, reporting test-set AUC and FNR by antibiotic class for both organisms. This analysis retains cultures up to (and including) the first non-susceptible event per patient × antibiotic class.

Both S2 and S3 reduce repeated, correlated observations within patients, although they do so in different ways. In both analyses, performance is consistently lower than baseline, with reduced AUC and higher FNR. Taken together, these results suggest that the main test set evaluation benefits from repeated patient-level observations and that the sensitivity analyses provide a more conservative estimate of performance for settings where repeat isolates are less common or where evaluation is restricted to earlier events.

**Sensitivity analysis S4: Restrict prior isolate history to the same specimen type**

We performed a post hoc sensitivity analysis to evaluate whether prior isolate history features should be restricted to the same specimen type as the index culture. We first identified the feature columns that represent prior isolate history. These included the recency features and the antibiotic exposure window features that encode day ranges. We then checked whether the dataset contained both the current specimen type for the index isolate and a column that indicates the specimen type of the most recent prior isolate.

When the prior specimen type column was available, we created masked versions of the training and test tables. For any row where the most recent prior specimen type differed from the current specimen type, we set the prior history feature values to zero (**Figure 4**). We then generated new predicted probabilities by scoring these masked features with the final trained model, without retraining. When the prior specimen-type column was unavailable, we performed a robustness check by stratifying the test set by current specimen type. We recomputed per-antibiotic metrics within each specimen-type stratum using the existing test predictions and thresholds. We saved these stratified results for reporting.

We assessed robustness across specimen types by reporting test performance separately for blood, urine, and other specimen categories. Across antibiotics, AUC values were generally similar across specimen types, suggesting that the model’s ability to distinguish non-susceptible from susceptible outcomes is broadly consistent across specimen sources. In contrast, FNR showed more variability across specimen types.

The most substantial differences tended to occur in strata with small numbers of non-susceptible cases, which was evident from the accompanying counts used to compute FNR. This is especially relevant for rare outcomes, such as Carbapenems non-susceptibility in certain specimen-type strata, where small changes in counts can produce large changes in FNR estimates. Overall, these stratified results support broadly comparable discrimination across specimen types, while indicating that threshold-based error rates can appear more variable in low-count strata and should be interpreted cautiously.


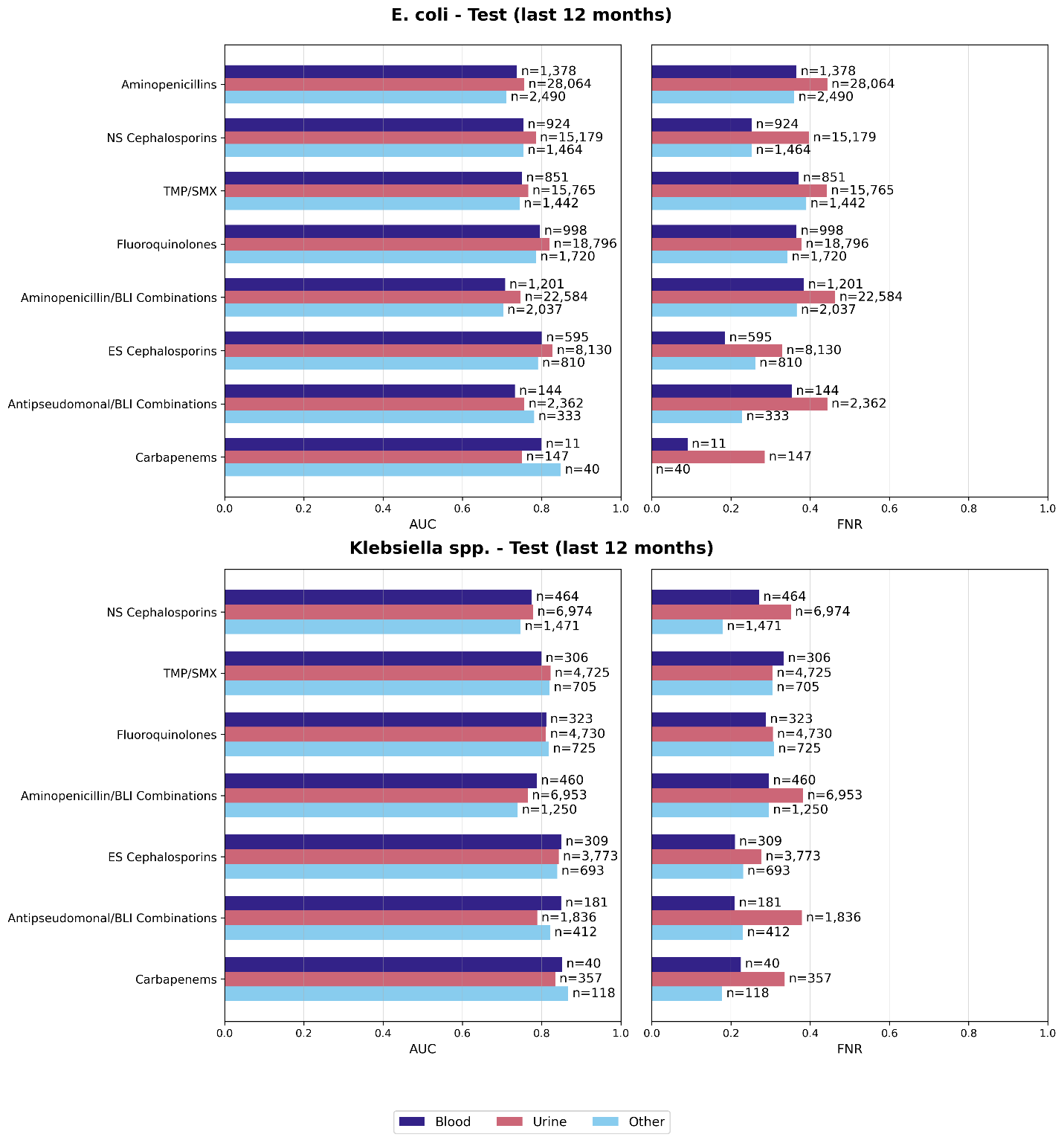


**Figure 4.** Horizontal bar plots showing test-set AUC and FNR by antibiotic class stratified by specimen type (blood, urine, other) for *E. coli* and *Klebsiella* spp., illustrating how discrimination and missed non-susceptible results vary by specimen source.

**Sensitivity analysis S5: Excluding very recent (1-3 day) antibiotic exposure features**

Antibiotic use in the 1-3 days before culture collection may partly reflect clinician suspicion and empiric prescribing. To test robustness, we re-scored the held-out 12-month test set after (i) setting all 1-3 day antibiotic exposure features to zero and (ii) permuting these features across observations, without retraining the model or changing the evaluation split. Performance changed only minimally across antibiotics in both organisms: for *E. coli*, the absolute change was ≤0.002 for AUC and ≤0.002 for AUPRC; for *Klebsiella* spp., the absolute change was ≤0.002 for AUC and ≤0.003 for AUPRC. Under permutation, changes remained small (AUC ≤0.005; AUPRC ≤0.005). Overall, these results suggest model performance is not primarily driven by very recent (1-3 day) antibiotic exposure signals.


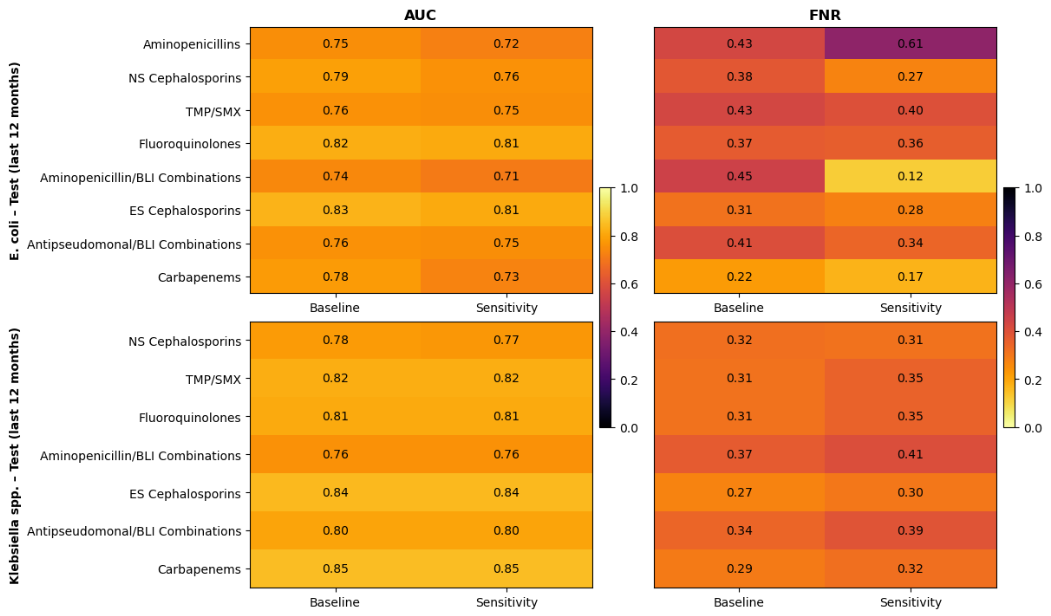


**Figure 5.** Heatmaps showing test-set AUC and FNR by antibiotic class for *E. coli* and *Klebsiella* spp. under the 1-3 day antibiotic-exposure sensitivity analysis, compared with the baseline model.

**Subgroup analyses: Sex, age group, and rurality**

We evaluated whether model performance was consistent across key patient subgroups. These analyses were post hoc and did not involve retraining. We used the same predicted probabilities and antibiotic-specific thresholds as in the main analysis and recomputed all metrics for each subgroup. Therefore, any differences across subgroups reflect differences in the subgroup data rather than differences caused by retraining.

We defined sex as male or female and rurality as urban or rural. We defined age groups using the continuous age variable with four bands: 18 to 44, 45 to 64, 65 to 74, and 75 and older. Missing ages were labeled Unknown.

We examined whether test set performance was consistent across key patient subgroups by recalculating metrics after stratifying the held-out data by rural versus urban residence, age group, and sex. In the plots below, each antibiotic is shown as a separate row. The left panels display the area under the ROC curve, which reflects how well the model ranks non-susceptible outcomes above susceptible outcomes across all possible thresholds, with higher values indicating better separation. The right panels show the FNR at the fixed antibiotic-specific operating thresholds used in the main analysis; lower values indicate fewer missed non-susceptible cases.

In the sex analysis, AUC values were broadly similar between females and males across both organisms, with some antibiotics showing modestly higher AUC among males (**Figure 6**). The more consistent differences were observed in FNR. For several antibiotics, females exhibited higher FNR than males, indicating that the same fixed operating thresholds resulted in a higher fraction of missed non-susceptible cases among females, even when overall discrimination was similar. This highlights that stable ranking performance does not necessarily imply equal threshold-based error rates across groups.


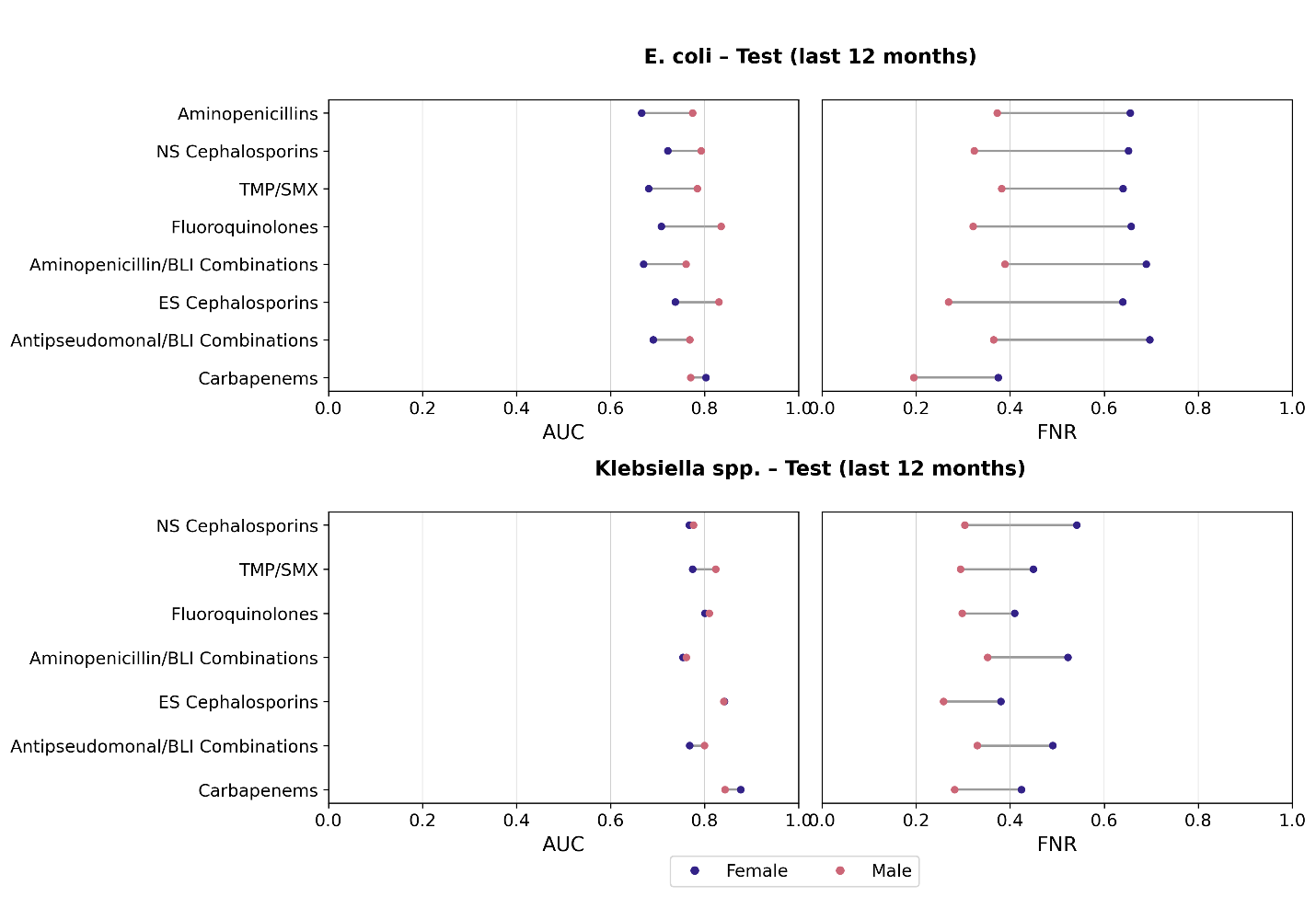


**Figure 6**. Dumbbell plots comparing test-set AUC and FNR between female and male patients for each antibiotic class and organism, evaluating whether discrimination and missed non-susceptible results differ by sex.

In the age-group analysis, patterns differed across organisms. For *E. coli*, younger adults tended to show lower AUC and higher FNR than older age groups across many antibiotics, indicating weaker separation and more missed non-susceptible cases at the same thresholds in the youngest stratum (**Figure 7**). Older groups, particularly the oldest age category, more often showed lower FNR, suggesting fewer missed non-susceptible cases at the fixed thresholds. For *Klebsiella* spp., age-related differences were less pronounced overall. AUC values were more tightly clustered across age groups, and FNRs were generally more similar across groups, although some antibiotics still showed noticeable variation.


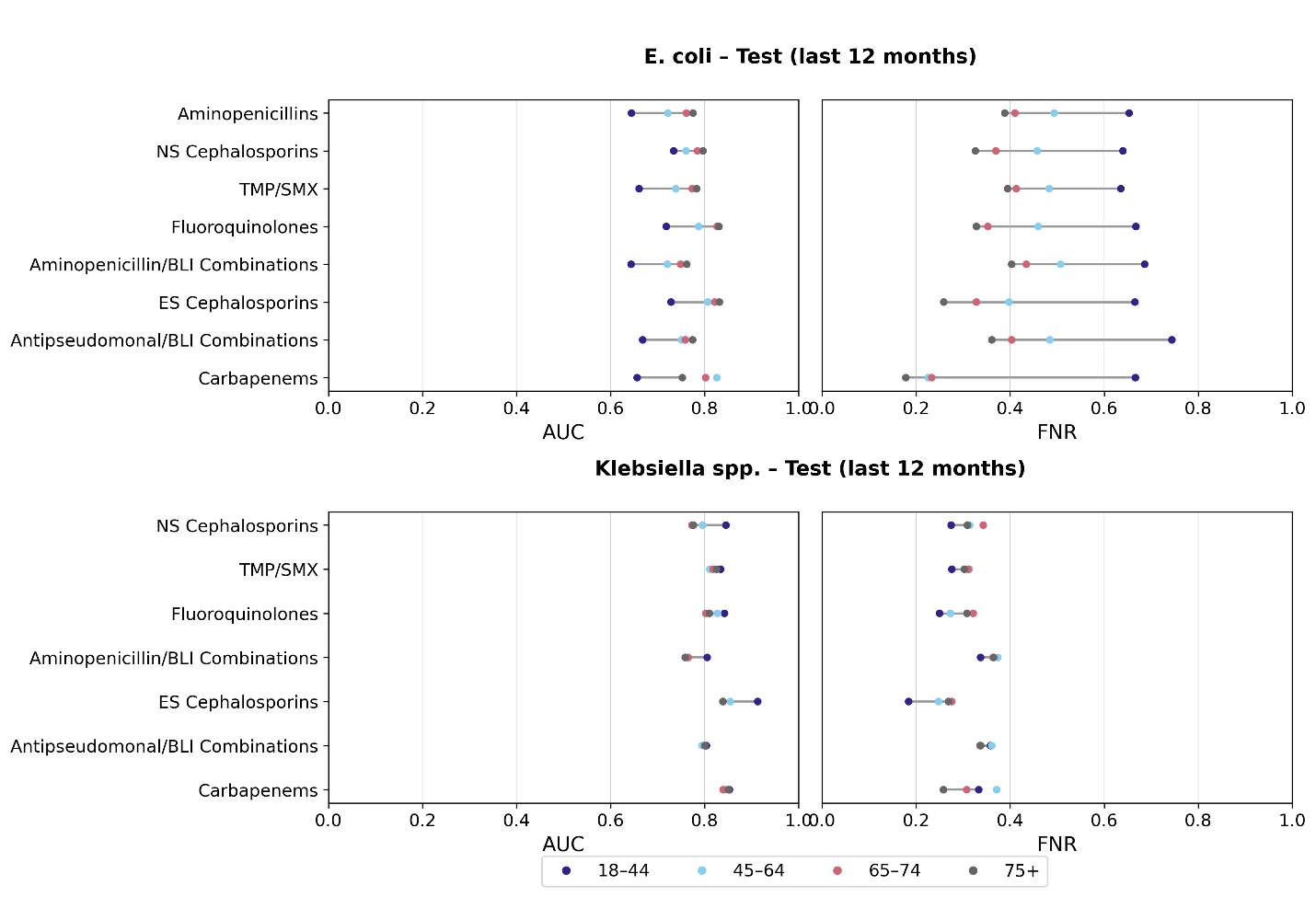


**Figure 7**. Dumbbell plots showing test-set AUC and FNR across age groups (18-44, 45-64, 65-74, 75+) for each antibiotic class and organism, assessing consistency of model performance across age strata.

In the rurality analysis, discrimination was generally similar between rural and urban patients. Across both organisms and most antibiotics, rural and urban AUC values were closely aligned, suggesting comparable ranking performance in the two groups (**Figure 8**). In contrast, FNR showed more separation. For several antibiotics, the rural group had higher FNR than the urban group, meaning that at the same operating thresholds, the model missed a larger fraction of non-susceptible cases among rural patients. The largest apparent gaps tended to occur for antibiotics with fewer non-susceptible cases, where FNR estimates can shift substantially with small changes in counts.


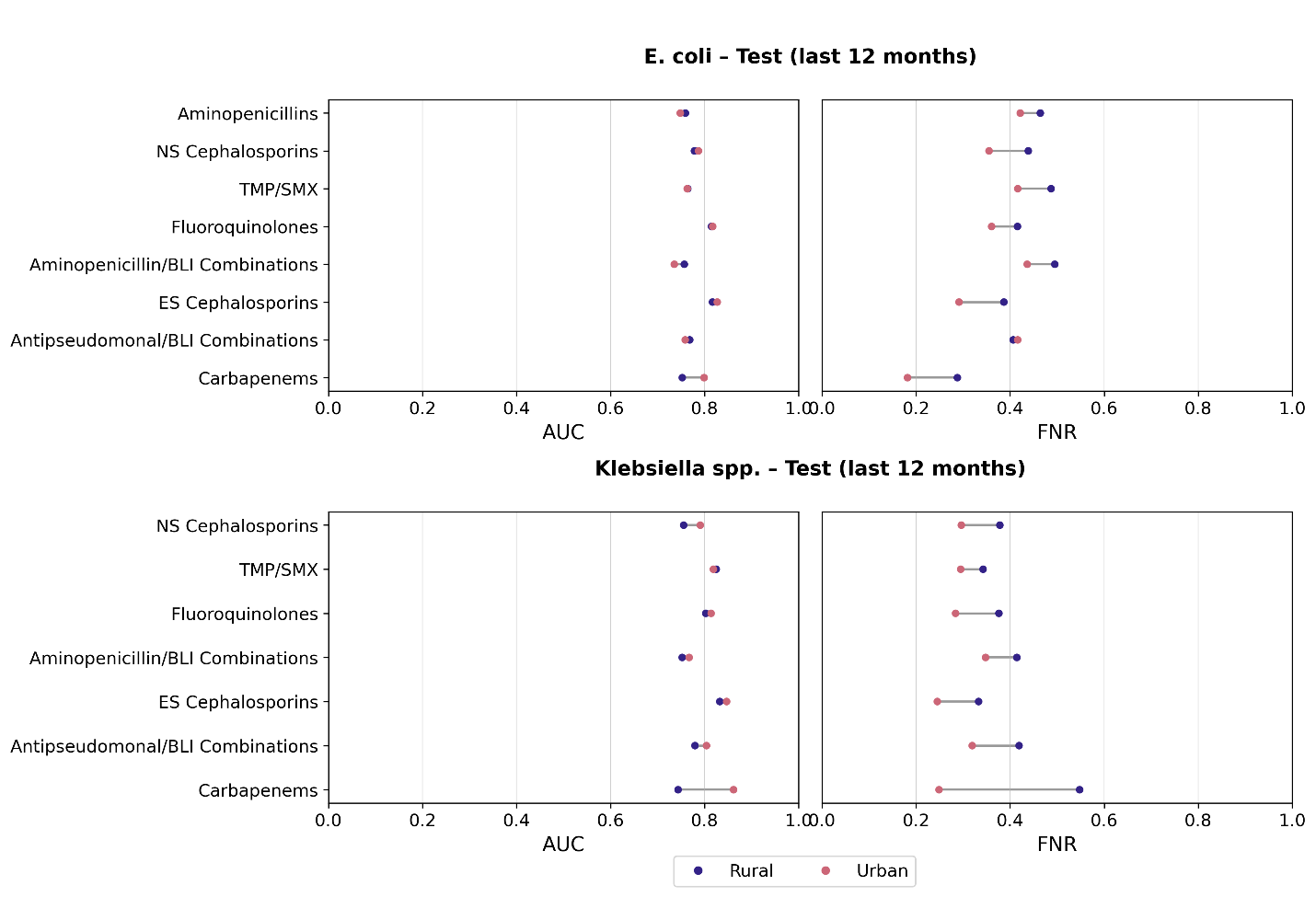


**Figure 8**. Dumbbell plots comparing test-set AUC and FNR between rural and urban patient residence groups for each antibiotic class and organism, highlighting whether performance differs by rurality.
