# Supplementary material for: Personalized Antibiogram: A Novel Multitask Machine Learning Framework for Simultaneous Prediction of Antimicrobial Resistance Profile With Enhanced Detection of Carbapenem Resistance in Enterobacteriaceae
Source: Clin Infect Dis. 2026 Jan 17;83(1):e1–9. doi: 10.1093/cid/ciag027 (PMC13393128; doi:10.1093/cid/ciag027)
Supplement: ciag027_Supplementary_Data [file ciag027_supplementary_data.zip › Supplementary Table 1 20260106.docx]

**Supplementary Table 1. Classifications of Antimicrobial Agents**

| Antimicrobial Groups | Antimicrobial Agents |
| --- | --- |
| Aminopenicillins | Ampicillin  Amoxicillin |
| Aminopenicillins/BLI Combinations | Ampicillin/Sulbactam  Amoxicillin/Clavulanate |
| Antipseudomonal Penicillins/BLI Combinations | Piperacillin/Tazobactam  Ticarcillin/Clavulanate |
| Narrow-Spectrum Cephalosporins | Cephalexin  Cefadroxil  Cefazolin  Cefuroxime  Cefaclor  Cefprozil |
| Extended-Spectrum Cephalosporins | Ceftriaxone  Cefotaxime  Ceftazidime  Cefepime |
| Carbapenems | Imipenem/Cilastatin  Meropenem  Ertapenem  Doripenem |
| Fluoroquinolones | Ofloxacin  Norfloxacin  Ciprofloxacin  Levofloxacin  Moxifloxacin  Gatifloxacin |
| Trimethoprim/Sulfamethoxazole | Trimethoprim/Sulfamethoxazole |

Abbreviations: BLI: Beta-lactamase inhibitor
