# Supplementary material for: Personalized Antibiogram: A Novel Multitask Machine Learning Framework for Simultaneous Prediction of Antimicrobial Resistance Profile With Enhanced Detection of Carbapenem Resistance in Enterobacteriaceae
Source: Clin Infect Dis. 2026 Jan 17;83(1):e1–9. doi: 10.1093/cid/ciag027 (PMC13393128; doi:10.1093/cid/ciag027)
Supplement: ciag027_Supplementary_Data [file ciag027_supplementary_data.zip › Supplementary Table 2 20260106.docx]

**Supplementary Table 2. List of Additional Patient-Level Variables**

| Variable | Description | *E. coli* Training | *E. coli* Test | *Klebsiella* Train | *Klebsiella* Test |
| --- | --- | --- | --- | --- | --- |
| n | Number of Unique Patients During Study Period | 287045 | 53913 | 139377 | 26907 |
| Clinical Classifications Software (CCS) for Procedures Performed | | | | | |
| CCS Category 1 | Incision and excision of CNS | 191 (0.1%) | 108 (0.2%) | 118 (0.1%) | 63 (0.2%) |
| CCS Category 2 | Insertion, replacement, or removal of extracranial ventricular shunt | 60 (0.0%) | 11 (0.0%) | 51 (0.0%) | 8 (0.0%) |
| CCS Category 3 | Laminectomy, excision intervertebral disc | 479 (0.2%) | 64 (0.1%) | 331 (0.2%) | 31 (0.1%) |
| CCS Category 4 | Diagnostic spinal tap | 404 (0.1%) | 38 (0.1%) | 253 (0.2%) | 35 (0.1%) |
| CCS Category 5 | Insertion of catheter or spinal stimulator and injection into spinal canal | 2110 (0.7%) | 328 (0.6%) | 1170 (0.8%) | 148 (0.6%) |
| CCS Category 6 | Decompression peripheral nerve | 340 (0.1%) | 49 (0.1%) | 171 (0.1%) | 21 (0.1%) |
| CCS Category 7 | Other diagnostic nervous system procedures | 3407 (1.2%) | 527 (1.0%) | 1695 (1.2%) | 309 (1.1%) |
| CCS Category 8 | Other non-OR or closed therapeutic nervous system procedures | 3531 (1.2%) | 671 (1.2%) | 2102 (1.5%) | 350 (1.3%) |
| CCS Category 9 | Other OR therapeutic nervous system procedures | 2652 (0.9%) | 645 (1.2%) | 1285 (0.9%) | 259 (1.0%) |
| CCS Category 10 | Thyroidectomy, partial or complete | 77 (0.0%) | 13 (0.0%) | 51 (0.0%) | 8 (0.0%) |
| CCS Category 11 | Diagnostic endocrine procedures | 772 (0.3%) | 500 (0.9%) | 483 (0.3%) | 350 (1.3%) |
| CCS Category 12 | Other therapeutic endocrine procedures | 173 (0.1%) | 56 (0.1%) | 91 (0.1%) | 31 (0.1%) |
| CCS Category 14 | Glaucoma procedures | 399 (0.1%) | 73 (0.1%) | 208 (0.1%) | 46 (0.2%) |
| CCS Category 15 | Lens and cataract procedures | 2579 (0.9%) | 459 (0.9%) | 1407 (1.0%) | 254 (0.9%) |
| CCS Category 16 | Repair of retinal tear, detachment | 97 (0.0%) | 11 (0.0%) | 64 (0.0%) | 8 (0.0%) |
| CCS Category 17 | Destruction of lesion of retina and choroid | 167 (0.1%) | 17 (0.0%) | 139 (0.1%) | 21 (0.1%) |
| CCS Category 18 | Diagnostic procedures on eye | 4052 (1.4%) | 673 (1.2%) | 2234 (1.6%) | 401 (1.5%) |
| CCS Category 19 | Other therapeutic procedures on eyelids, conjunctiva, cornea | 969 (0.3%) | 145 (0.3%) | 501 (0.4%) | 84 (0.3%) |
| CCS Category 20 | Other intraocular therapeutic procedures | 1801 (0.6%) | 348 (0.6%) | 1101 (0.8%) | 210 (0.8%) |
| CCS Category 21 | Other extraocular muscle and orbit therapeutic procedures | 47 (0.0%) | 8 (0.0%) | 21 (0.0%) | 1 (0.0%) |
| CCS Category 23 | Myringotomy | 121 (0.0%) | 15 (0.0%) | 76 (0.1%) | 16 (0.1%) |
| CCS Category 25 | Diagnostic procedures on ear | 364 (0.1%) | 50 (0.1%) | 176 (0.1%) | 26 (0.1%) |
| CCS Category 26 | Other therapeutic ear procedures | 5167 (1.8%) | 1023 (1.9%) | 2849 (2.0%) | 605 (2.2%) |
| CCS Category 27 | Control of epistaxis | 143 (0.0%) | 22 (0.0%) | 102 (0.1%) | 16 (0.1%) |
| CCS Category 28 | Plastic procedures on nose | 177 (0.1%) | 42 (0.1%) | 95 (0.1%) | 31 (0.1%) |
| CCS Category 29 | Oral and Dental Services | 71 (0.0%) | 7 (0.0%) | 52 (0.0%) | 8 (0.0%) |
| CCS Category 31 | Diagnostic procedures on nose, mouth and pharynx | 16994 (5.9%) | 2926 (5.4%) | 12475 (9.0%) | 2342 (8.7%) |
| CCS Category 33 | Other OR therapeutic procedures on nose, mouth and pharynx | 2232 (0.8%) | 365 (0.7%) | 1689 (1.2%) | 292 (1.1%) |
| CCS Category 34 | Tracheostomy, temporary and permanent | 692 (0.2%) | 75 (0.1%) | 846 (0.6%) | 100 (0.4%) |
| CCS Category 35 | Tracheoscopy and laryngoscopy with biopsy | 2837 (1.0%) | 471 (0.9%) | 2145 (1.5%) | 361 (1.3%) |
| CCS Category 36 | Lobectomy or pneumonectomy | 238 (0.1%) | 21 (0.0%) | 204 (0.1%) | 27 (0.1%) |
| CCS Category 37 | Diagnostic bronchoscopy and biopsy of bronchus | 1516 (0.5%) | 200 (0.4%) | 1595 (1.1%) | 211 (0.8%) |
| CCS Category 38 | Other diagnostic procedures on lung and bronchus | 9584 (3.3%) | 1554 (2.9%) | 6100 (4.4%) | 1067 (4.0%) |
| CCS Category 39 | Incision of pleura, thoracentesis, chest drainage | 1112 (0.4%) | 145 (0.3%) | 1091 (0.8%) | 162 (0.6%) |
| CCS Category 40 | Other diagnostic procedures of respiratory tract and mediastinum | 130 (0.0%) | 45 (0.1%) | 103 (0.1%) | 28 (0.1%) |
| CCS Category 41 | Other non-OR therapeutic procedures on respiratory system | 10493 (3.7%) | 1693 (3.1%) | 8137 (5.8%) | 1432 (5.3%) |
| CCS Category 42 | Other OR therapeutic procedures on respiratory system | 1454 (0.5%) | 201 (0.4%) | 1582 (1.1%) | 234 (0.9%) |
| CCS Category 43 | Heart valve procedures | 254 (0.1%) | 40 (0.1%) | 250 (0.2%) | 19 (0.1%) |
| CCS Category 44 | Coronary artery bypass graft (CABG) | 349 (0.1%) | 40 (0.1%) | 406 (0.3%) | 46 (0.2%) |
| CCS Category 45 | Percutaneous transluminal coronary angioplasty (PTCA) | 586 (0.2%) | 64 (0.1%) | 441 (0.3%) | 48 (0.2%) |
| CCS Category 47 | Diagnostic cardiac catheterization, coronary arteriography | 2857 (1.0%) | 477 (0.9%) | 2250 (1.6%) | 374 (1.4%) |
| CCS Category 48 | Insertion, revision, replacement, removal of cardiac pacemaker or cardioverter/defibrillator | 760 (0.3%) | 120 (0.2%) | 604 (0.4%) | 93 (0.3%) |
| CCS Category 49 | Other OR heart procedures | 278 (0.1%) | 48 (0.1%) | 236 (0.2%) | 41 (0.2%) |
| CCS Category 50 | Extracorporeal circulation auxiliary to open heart procedures | - | - | 49 (0.0%) | 12 (0.0%) |
| CCS Category 51 | Endarterectomy, vessel of head and neck | 158 (0.1%) | 18 (0.0%) | 115 (0.1%) | 21 (0.1%) |
| CCS Category 52 | Aortic resection, replacement or anastomosis | 153 (0.1%) | 21 (0.0%) | 122 (0.1%) | 19 (0.1%) |
| CCS Category 54 | Other vascular catheterization, not heart | 31815 (11.1%) | 5176 (9.6%) | 21204 (15.2%) | 3631 (13.5%) |
| CCS Category 55 | Peripheral vascular bypass | 512 (0.2%) | 66 (0.1%) | 436 (0.3%) | 67 (0.2%) |
| CCS Category 56 | Other vascular bypass and shunt, not heart | 41 (0.0%) | 12 (0.0%) | 40 (0.0%) | 15 (0.1%) |
| CCS Category 57 | Creation, revision and removal of arteriovenous fistula or vessel-to-vessel cannula for dialysis | 850 (0.3%) | 109 (0.2%) | 776 (0.6%) | 92 (0.3%) |
| CCS Category 58 | Hemodialysis | 1823 (0.6%) | 241 (0.4%) | 1860 (1.3%) | 253 (0.9%) |
| CCS Category 59 | Other OR procedures on vessels of head and neck | 175 (0.1%) | 17 (0.0%) | 136 (0.1%) | 19 (0.1%) |
| CCS Category 60 | Embolectomy and endarterectomy of lower limbs | 436 (0.2%) | 73 (0.1%) | 372 (0.3%) | 60 (0.2%) |
| CCS Category 61 | Other OR procedures on vessels other than head and neck | 3788 (1.3%) | 475 (0.9%) | 3096 (2.2%) | 406 (1.5%) |
| CCS Category 62 | Other diagnostic cardiovascular procedures | 12350 (4.3%) | 2093 (3.9%) | 8862 (6.4%) | 1592 (5.9%) |
| CCS Category 63 | Other non-OR therapeutic cardiovascular procedures | 5073 (1.8%) | 752 (1.4%) | 3935 (2.8%) | 644 (2.4%) |
| CCS Category 65 | Bone marrow biopsy | 608 (0.2%) | 84 (0.2%) | 377 (0.3%) | 56 (0.2%) |
| CCS Category 66 | Procedures on spleen | 32 (0.0%) | 4 (0.0%) | 29 (0.0%) | 2 (0.0%) |
| CCS Category 67 | Other therapeutic procedures, hemic and lymphatic system | 1027 (0.4%) | 137 (0.3%) | 842 (0.6%) | 107 (0.4%) |
| CCS Category 68 | Injection or ligation of esophageal varices | 134 (0.0%) | 18 (0.0%) | 89 (0.1%) | 14 (0.1%) |
| CCS Category 69 | Esophageal dilatation | 482 (0.2%) | 99 (0.2%) | 323 (0.2%) | 53 (0.2%) |
| CCS Category 70 | Upper gastrointestinal endoscopy, biopsy | 7780 (2.7%) | 1133 (2.1%) | 5075 (3.6%) | 829 (3.1%) |
| CCS Category 71 | Gastrostomy, temporary and permanent | 1545 (0.5%) | 186 (0.3%) | 1638 (1.2%) | 223 (0.8%) |
| CCS Category 72 | Colostomy, temporary and permanent | 468 (0.2%) | 53 (0.1%) | 288 (0.2%) | 45 (0.2%) |
| CCS Category 73 | Ileostomy and other enterostomy | 596 (0.2%) | 55 (0.1%) | 618 (0.4%) | 62 (0.2%) |
| CCS Category 74 | Gastrectomy, partial and total | 49 (0.0%) | 6 (0.0%) | 52 (0.0%) | 2 (0.0%) |
| CCS Category 75 | Small bowel resection | 557 (0.2%) | 63 (0.1%) | 438 (0.3%) | 52 (0.2%) |
| CCS Category 76 | Colonoscopy and biopsy | 8796 (3.1%) | 1240 (2.3%) | 4462 (3.2%) | 717 (2.7%) |
| CCS Category 77 | Proctoscopy and anorectal biopsy | 1315 (0.5%) | 166 (0.3%) | 692 (0.5%) | 109 (0.4%) |
| CCS Category 78 | Colorectal resection | 1838 (0.6%) | 180 (0.3%) | 959 (0.7%) | 94 (0.3%) |
| CCS Category 80 | Appendectomy | 577 (0.2%) | 71 (0.1%) | 150 (0.1%) | 16 (0.1%) |
| CCS Category 81 | Hemorrhoid procedures | 210 (0.1%) | 24 (0.0%) | 87 (0.1%) | 11 (0.0%) |
| CCS Category 82 | Endoscopic retrograde cannulation of pancreas (ERCP) | 886 (0.3%) | 133 (0.2%) | 873 (0.6%) | 134 (0.5%) |
| CCS Category 83 | Biopsy of liver | 522 (0.2%) | 84 (0.2%) | 393 (0.3%) | 56 (0.2%) |
| CCS Category 84 | Cholecystectomy and common duct exploration | 1155 (0.4%) | 168 (0.3%) | 813 (0.6%) | 129 (0.5%) |
| CCS Category 85 | Inguinal and femoral hernia repair | 530 (0.2%) | 84 (0.2%) | 406 (0.3%) | 61 (0.2%) |
| CCS Category 86 | Other hernia repair | 642 (0.2%) | 79 (0.1%) | 419 (0.3%) | 52 (0.2%) |
| CCS Category 87 | Laparoscopy | 289 (0.1%) | 34 (0.1%) | 224 (0.2%) | 35 (0.1%) |
| CCS Category 88 | Abdominal paracentesis | 1044 (0.4%) | 145 (0.3%) | 790 (0.6%) | 127 (0.5%) |
| CCS Category 89 | Exploratory laparotomy | 425 (0.1%) | 46 (0.1%) | 326 (0.2%) | 35 (0.1%) |
| CCS Category 90 | Excision, lysis peritoneal adhesions | 156 (0.1%) | 11 (0.0%) | 153 (0.1%) | 13 (0.0%) |
| CCS Category 91 | Peritoneal dialysis | 2110 (0.7%) | 322 (0.6%) | 2115 (1.5%) | 313 (1.2%) |
| CCS Category 92 | Other bowel diagnostic procedures | 25 (0.0%) | 2 (0.0%) | 16 (0.0%) | 1 (0.0%) |
| CCS Category 93 | Other non-OR upper GI therapeutic procedures | 51 (0.0%) | 7 (0.0%) | 39 (0.0%) | 12 (0.0%) |
| CCS Category 94 | Other OR upper GI therapeutic procedures | 1185 (0.4%) | 364 (0.7%) | 878 (0.6%) | 275 (1.0%) |
| CCS Category 95 | Other non-OR lower GI therapeutic procedures | 63 (0.0%) | 5 (0.0%) | 22 (0.0%) | 4 (0.0%) |
| CCS Category 96 | Other OR lower GI therapeutic procedures | 1632 (0.6%) | 206 (0.4%) | 690 (0.5%) | 112 (0.4%) |
| CCS Category 97 | Other gastrointestinal diagnostic procedures | 1580 (0.6%) | 303 (0.6%) | 872 (0.6%) | 172 (0.6%) |
| CCS Category 98 | Other non-OR gastrointestinal therapeutic procedures | 487 (0.2%) | 68 (0.1%) | 434 (0.3%) | 50 (0.2%) |
| CCS Category 99 | Other OR gastrointestinal therapeutic procedures | 3631 (1.3%) | 506 (0.9%) | 2464 (1.8%) | 356 (1.3%) |
| CCS Category 100 | Endoscopy and endoscopic biopsy of the urinary tract | 12532 (4.4%) | 2157 (4.0%) | 8255 (5.9%) | 1472 (5.5%) |
| CCS Category 101 | Transurethral excision, drainage, or removal urinary obstruction | 2910 (1.0%) | 441 (0.8%) | 2349 (1.7%) | 353 (1.3%) |
| CCS Category 102 | Ureteral catheterization | 2357 (0.8%) | 358 (0.7%) | 1594 (1.1%) | 251 (0.9%) |
| CCS Category 103 | Nephrotomy and nephrostomy | 1625 (0.6%) | 294 (0.5%) | 1555 (1.1%) | 284 (1.1%) |
| CCS Category 104 | Nephrectomy, partial or complete | 245 (0.1%) | 28 (0.1%) | 189 (0.1%) | 29 (0.1%) |
| CCS Category 105 | Kidney transplant | 63 (0.0%) | 15 (0.0%) | 46 (0.0%) | 8 (0.0%) |
| CCS Category 106 | Genitourinary incontinence procedures | 169 (0.1%) | 16 (0.0%) | 72 (0.1%) | 14 (0.1%) |
| CCS Category 107 | Extracorporeal lithotripsy, urinary | 1058 (0.4%) | 161 (0.3%) | 635 (0.5%) | 127 (0.5%) |
| CCS Category 108 | Indwelling catheter | 15616 (5.4%) | 2758 (5.1%) | 13012 (9.3%) | 2383 (8.9%) |
| CCS Category 109 | Procedures on the urethra | 1134 (0.4%) | 174 (0.3%) | 779 (0.6%) | 98 (0.4%) |
| CCS Category 110 | Other diagnostic procedures of urinary tract | 701 (0.2%) | 92 (0.2%) | 474 (0.3%) | 69 (0.3%) |
| CCS Category 111 | Other non-OR therapeutic procedures of urinary tract | 4925 (1.7%) | 983 (1.8%) | 4486 (3.2%) | 949 (3.5%) |
| CCS Category 112 | Other OR therapeutic procedures of urinary tract | 2580 (0.9%) | 403 (0.7%) | 2014 (1.4%) | 325 (1.2%) |
| CCS Category 113 | Transurethral resection of prostate (TURP) | 891 (0.3%) | 142 (0.3%) | 684 (0.5%) | 132 (0.5%) |
| CCS Category 114 | Open prostatectomy | 1303 (0.5%) | 151 (0.3%) | 1086 (0.8%) | 136 (0.5%) |
| CCS Category 115 | Circumcision | 135 (0.0%) | 22 (0.0%) | 81 (0.1%) | 11 (0.0%) |
| CCS Category 116 | Diagnostic procedures, male genital | 3156 (1.1%) | 358 (0.7%) | 926 (0.7%) | 136 (0.5%) |
| CCS Category 117 | Other non-OR therapeutic procedures, male genital | 405 (0.1%) | 51 (0.1%) | 166 (0.1%) | 13 (0.0%) |
| CCS Category 118 | Other OR therapeutic procedures, male genital | 847 (0.3%) | 112 (0.2%) | 481 (0.3%) | 83 (0.3%) |
| CCS Category 119 | Oophorectomy; unilateral and bilateral | 65 (0.0%) | 11 (0.0%) | 20 (0.0%) | 1 (0.0%) |
| CCS Category 124 | Hysterectomy, abdominal and vaginal | 184 (0.1%) | 22 (0.0%) | 39 (0.0%) | 2 (0.0%) |
| CCS Category 125 | Other excision of cervix and uterus | 113 (0.0%) | 22 (0.0%) | 18 (0.0%) | 2 (0.0%) |
| CCS Category 129 | Repair of cystocele and rectocele, obliteration of vaginal vault | 41 (0.0%) | 6 (0.0%) | 10 (0.0%) | 4 (0.0%) |
| CCS Category 130 | Other diagnostic procedures, female organs | 4460 (1.6%) | 446 (0.8%) | 845 (0.6%) | 91 (0.3%) |
| CCS Category 131 | Other non-OR therapeutic procedures, female organs | 645 (0.2%) | 106 (0.2%) | 103 (0.1%) | 9 (0.0%) |
| CCS Category 132 | Other OR therapeutic procedures, female organs | 190 (0.1%) | 38 (0.1%) | 42 (0.0%) | 9 (0.0%) |
| CCS Category 142 | Partial excision bone | 970 (0.3%) | 115 (0.2%) | 770 (0.6%) | 102 (0.4%) |
| CCS Category 143 | Bunionectomy or repair of toe deformities | 157 (0.1%) | 28 (0.1%) | 79 (0.1%) | 12 (0.0%) |
| CCS Category 144 | Treatment, facial fracture or dislocation | 44 (0.0%) | 8 (0.0%) | 33 (0.0%) | 6 (0.0%) |
| CCS Category 145 | Treatment, fracture or dislocation of radius and ulna | 69 (0.0%) | 9 (0.0%) | 21 (0.0%) | 4 (0.0%) |
| CCS Category 146 | Treatment, fracture or dislocation of hip and femur | 577 (0.2%) | 72 (0.1%) | 379 (0.3%) | 59 (0.2%) |
| CCS Category 147 | Treatment, fracture or dislocation of lower extremity (other than hip or femur) | 218 (0.1%) | 33 (0.1%) | 112 (0.1%) | 22 (0.1%) |
| CCS Category 148 | Other fracture and dislocation procedure | 183 (0.1%) | 24 (0.0%) | 107 (0.1%) | 14 (0.1%) |
| CCS Category 149 | Arthroscopy | 51 (0.0%) | 15 (0.0%) | 28 (0.0%) | 2 (0.0%) |
| CCS Category 151 | Excision of semilunar cartilage of knee | 60 (0.0%) | 10 (0.0%) | 23 (0.0%) | 3 (0.0%) |
| CCS Category 152 | Arthroplasty knee | 544 (0.2%) | 101 (0.2%) | 235 (0.2%) | 39 (0.1%) |
| CCS Category 153 | Hip replacement, total and partial | 429 (0.1%) | 65 (0.1%) | 271 (0.2%) | 27 (0.1%) |
| CCS Category 154 | Arthroplasty other than hip or knee | 218 (0.1%) | 34 (0.1%) | 110 (0.1%) | 15 (0.1%) |
| CCS Category 155 | Arthrocentesis | 8833 (3.1%) | 1764 (3.3%) | 3950 (2.8%) | 809 (3.0%) |
| CCS Category 156 | Injections and aspirations of muscles, tendons, bursa, joints and soft tissue | 1740 (0.6%) | 339 (0.6%) | 677 (0.5%) | 131 (0.5%) |
| CCS Category 157 | Amputation of lower extremity | 2730 (1.0%) | 398 (0.7%) | 2559 (1.8%) | 384 (1.4%) |
| CCS Category 158 | Spinal fusion | 341 (0.1%) | 50 (0.1%) | 247 (0.2%) | 32 (0.1%) |
| CCS Category 159 | Other diagnostic procedures on musculoskeletal system | 1592 (0.6%) | 195 (0.4%) | 1148 (0.8%) | 148 (0.6%) |
| CCS Category 160 | Other therapeutic procedures on muscles and tendons | 1180 (0.4%) | 180 (0.3%) | 762 (0.5%) | 125 (0.5%) |
| CCS Category 161 | Other OR therapeutic procedures on bone | 652 (0.2%) | 89 (0.2%) | 492 (0.4%) | 58 (0.2%) |
| CCS Category 162 | Other OR therapeutic procedures on joints | 441 (0.2%) | 64 (0.1%) | 289 (0.2%) | 29 (0.1%) |
| CCS Category 163 | Other non-OR therapeutic procedures on musculoskeletal system | 2321 (0.8%) | 620 (1.2%) | 704 (0.5%) | 177 (0.7%) |
| CCS Category 164 | Other OR therapeutic procedures on musculoskeletal system | 219 (0.1%) | 36 (0.1%) | 190 (0.1%) | 24 (0.1%) |
| CCS Category 165 | Breast biopsy and other diagnostic procedures on breast | 171 (0.1%) | 26 (0.0%) | 48 (0.0%) | 8 (0.0%) |
| CCS Category 166 | Lumpectomy, quadrantectomy of breast | 57 (0.0%) | 12 (0.0%) | 14 (0.0%) | 1 (0.0%) |
| CCS Category 167 | Mastectomy | - | - | 16 (0.0%) | 5 (0.0%) |
| CCS Category 168 | Incision and drainage, skin and subcutaneous tissue | 2369 (0.8%) | 283 (0.5%) | 1613 (1.2%) | 226 (0.8%) |
| CCS Category 169 | Debridement of wound, infection or burn | 7962 (2.8%) | 1234 (2.3%) | 6398 (4.6%) | 1090 (4.1%) |
| CCS Category 170 | Excision of skin lesion | 22342 (7.8%) | 3998 (7.4%) | 13035 (9.4%) | 2438 (9.1%) |
| CCS Category 171 | Suture of skin and subcutaneous tissue | 1902 (0.7%) | 328 (0.6%) | 1310 (0.9%) | 191 (0.7%) |
| CCS Category 172 | Skin graft | 1735 (0.6%) | 235 (0.4%) | 1422 (1.0%) | 199 (0.7%) |
| CCS Category 173 | Other diagnostic procedures on skin and subcutaneous tissue | 2316 (0.8%) | 56 (0.1%) | 1361 (1.0%) | 36 (0.1%) |
| CCS Category 174 | Other non-OR therapeutic procedures on skin and breast | 33489 (11.7%) | 7257 (13.5%) | 20471 (14.7%) | 4650 (17.3%) |
| CCS Category 175 | Other OR therapeutic procedures on skin and breast | 503 (0.2%) | 68 (0.1%) | 282 (0.2%) | 47 (0.2%) |
| CCS Category 176 | Other organ transplantation | 30 (0.0%) | 3 (0.0%) | 23 (0.0%) | 5 (0.0%) |
| CCS Category 177 | Computerized axial tomography (CT) scan head | 33556 (11.7%) | 6023 (11.2%) | 21323 (15.3%) | 3970 (14.8%) |
| CCS Category 178 | CT scan chest | 33572 (11.7%) | 6485 (12.0%) | 23150 (16.6%) | 4514 (16.8%) |
| CCS Category 179 | CT scan abdomen | 57306 (20.0%) | 10600 (19.7%) | 34300 (24.6%) | 6763 (25.1%) |
| CCS Category 180 | Other CT scan | 17387 (6.1%) | 3396 (6.3%) | 11325 (8.1%) | 2343 (8.7%) |
| CCS Category 181 | Myelogram | 90 (0.0%) | 15 (0.0%) | 71 (0.1%) | 7 (0.0%) |
| CCS Category 182 | Mammography | 5358 (1.9%) | 1029 (1.9%) | 1302 (0.9%) | 216 (0.8%) |
| CCS Category 183 | Routine chest X-ray | 92016 (32.1%) | 14850 (27.5%) | 58694 (42.1%) | 10000 (37.2%) |
| CCS Category 184 | Intraoperative cholangiogram | 103 (0.0%) | 11 (0.0%) | 90 (0.1%) | 12 (0.0%) |
| CCS Category 185 | Upper gastrointestinal X-ray | 5067 (1.8%) | 822 (1.5%) | 3733 (2.7%) | 634 (2.4%) |
| CCS Category 186 | Lower gastrointestinal X-ray | 261 (0.1%) | 28 (0.1%) | 175 (0.1%) | 17 (0.1%) |
| CCS Category 187 | Intravenous pyelogram | 168 (0.1%) | 31 (0.1%) | 105 (0.1%) | 15 (0.1%) |
| CCS Category 189 | Contrast aortogram | 1890 (0.7%) | 309 (0.6%) | 1634 (1.2%) | 263 (1.0%) |
| CCS Category 190 | Contrast arteriogram of femoral and lower extremity arteries | 240 (0.1%) | 32 (0.1%) | 192 (0.1%) | 23 (0.1%) |
| CCS Category 191 | Arterio- or venogram (not heart and head) | 4256 (1.5%) | 771 (1.4%) | 3276 (2.4%) | 600 (2.2%) |
| CCS Category 192 | Diagnostic ultrasound of head and neck | 9908 (3.5%) | 1584 (2.9%) | 5648 (4.1%) | 919 (3.4%) |
| CCS Category 193 | Diagnostic ultrasound of heart (echocardiogram) | 28539 (9.9%) | 4723 (8.8%) | 20487 (14.7%) | 3627 (13.5%) |
| CCS Category 195 | Diagnostic ultrasound of urinary tract | 188 (0.1%) | 30 (0.1%) | 190 (0.1%) | 48 (0.2%) |
| CCS Category 196 | Diagnostic ultrasound of abdomen or retroperitoneum | 33573 (11.7%) | 5516 (10.2%) | 21672 (15.5%) | 3714 (13.8%) |
| CCS Category 197 | Other diagnostic ultrasound | 37778 (13.2%) | 6201 (11.5%) | 23654 (17.0%) | 4057 (15.1%) |
| CCS Category 198 | Magnetic resonance imaging | 26316 (9.2%) | 4587 (8.5%) | 14924 (10.7%) | 2795 (10.4%) |
| CCS Category 199 | Electroencephalogram (EEG) | 2358 (0.8%) | 302 (0.6%) | 1842 (1.3%) | 246 (0.9%) |
| CCS Category 200 | Nonoperative urinary system measurements | 149114 (51.9%) | 26279 (48.7%) | 66923 (48.0%) | 12237 (45.5%) |
| CCS Category 201 | Cardiac stress tests | 5639 (2.0%) | 807 (1.5%) | 3249 (2.3%) | 487 (1.8%) |
| CCS Category 202 | Electrocardiogram | 92958 (32.4%) | 17890 (33.2%) | 57844 (41.5%) | 11507 (42.8%) |
| CCS Category 203 | Electrographic cardiac monitoring | 11197 (3.9%) | 2235 (4.1%) | 6655 (4.8%) | 1415 (5.3%) |
| CCS Category 204 | Swan-Ganz catheterization for monitoring | 302 (0.1%) | 28 (0.1%) | 351 (0.3%) | 37 (0.1%) |
| CCS Category 205 | Arterial blood gases | 17923 (6.2%) | 3680 (6.8%) | 13421 (9.6%) | 2737 (10.2%) |
| CCS Category 206 | Microscopic examination (bacterial smear, culture, toxicology) | 238016 (82.9%) | 44243 (82.1%) | 113566 (81.5%) | 21884 (81.3%) |
| CCS Category 207 | Radioisotope bone scan | 2905 (1.0%) | 230 (0.4%) | 1820 (1.3%) | 144 (0.5%) |
| CCS Category 208 | Radioisotope pulmonary scan | 879 (0.3%) | 104 (0.2%) | 669 (0.5%) | 91 (0.3%) |
| CCS Category 209 | Radioisotope scan and function studies | 13466 (4.7%) | 2135 (4.0%) | 9074 (6.5%) | 1492 (5.5%) |
| CCS Category 210 | Other radioisotope scan | 467 (0.2%) | 44 (0.1%) | 380 (0.3%) | 36 (0.1%) |
| CCS Category 211 | Therapeutic radiology | 1525 (0.5%) | 216 (0.4%) | 1014 (0.7%) | 146 (0.5%) |
| CCS Category 212 | Diagnostic physical, occupational, and speech therapy | 57661 (20.1%) | 10948 (20.3%) | 38287 (27.5%) | 7444 (27.7%) |
| CCS Category 213 | Physical, occupational, and speech therapy exercises; manipulation; and other procedures | 55868 (19.5%) | 10660 (19.8%) | 35395 (25.4%) | 6777 (25.2%) |
| CCS Category 214 | Traction, splints, and other wound care | 12989 (4.5%) | 1902 (3.5%) | 9914 (7.1%) | 1609 (6.0%) |
| CCS Category 215 | Other physical, occupational, and speech therapy and rehabilitation | 55969 (19.5%) | 10535 (19.5%) | 35866 (25.7%) | 6963 (25.9%) |
| CCS Category 216 | Respiratory intubation and mechanical ventilation | 10948 (3.8%) | 1992 (3.7%) | 7250 (5.2%) | 1431 (5.3%) |
| CCS Category 217 | Other respiratory therapy | 7188 (2.5%) | 1185 (2.2%) | 5724 (4.1%) | 1028 (3.8%) |
| CCS Category 218 | Psychological and psychiatric evaluation and therapy | 83378 (29.0%) | 16212 (30.1%) | 44758 (32.1%) | 9366 (34.8%) |
| CCS Category 219 | Alcohol and drug management, treatment, and rehabilitation | 1382 (0.5%) | 211 (0.4%) | 786 (0.6%) | 163 (0.6%) |
| CCS Category 220 | Ophthalmologic and otologic diagnosis and treatment | 73438 (25.6%) | 13526 (25.1%) | 37861 (27.2%) | 7329 (27.2%) |
| CCS Category 221 | Nasogastric tube | 160 (0.1%) | 19 (0.0%) | 157 (0.1%) | 19 (0.1%) |
| CCS Category 222 | Blood and blood product transfusion | 1271 (0.4%) | 148 (0.3%) | 976 (0.7%) | 124 (0.5%) |
| CCS Category 223 | Enteral and parenteral nutrition | 18 (0.0%) | 5 (0.0%) | 18 (0.0%) | 6 (0.0%) |
| CCS Category 224 | Cancer chemotherapy | 6408 (2.2%) | 1123 (2.1%) | 4634 (3.3%) | 831 (3.1%) |
| CCS Category 225 | Conversion of cardiac rhythm | 255 (0.1%) | 47 (0.1%) | 167 (0.1%) | 28 (0.1%) |
| CCS Category 226 | Other diagnostic radiology and related techniques | 85707 (29.9%) | 14193 (26.3%) | 50126 (36.0%) | 8561 (31.8%) |
| CCS Category 227 | Consultation, evaluation, and preventative care | 271688 (94.6%) | 50971 (94.5%) | 133652 (95.9%) | 25778 (95.8%) |
| CCS Category 228 | Prophylactic vaccinations and inoculations | 75820 (26.4%) | 12568 (23.3%) | 37591 (27.0%) | 6582 (24.5%) |
| CCS Category 229 | Nonoperative removal of foreign body | 324 (0.1%) | 52 (0.1%) | 186 (0.1%) | 36 (0.1%) |
| CCS Category 230 | Extracorporeal shock wave, other than urinary | 23 (0.0%) | 4 (0.0%) | 21 (0.0%) | 2 (0.0%) |
| CCS Category 231 | Other therapeutic procedures | 59862 (20.9%) | 10264 (19.0%) | 37999 (27.3%) | 6826 (25.4%) |
| CCS Category 232 | Anesthesia | 28039 (9.8%) | 4468 (8.3%) | 18775 (13.5%) | 3064 (11.4%) |
| CCS Category 233 | Laboratory - Chemistry and Hematology | 249664 (87.0%) | 46448 (86.2%) | 124808 (89.5%) | 23913 (88.9%) |
| CCS Category 234 | Pathology | 56719 (19.8%) | 9237 (17.1%) | 31440 (22.6%) | 5370 (20.0%) |
| CCS Category 235 | Other Laboratory | 176615 (61.5%) | 31075 (57.6%) | 86269 (61.9%) | 15809 (58.8%) |
| CCS Category 236 | Nonhospital-based care (e.g., home health care, hospice) | 6127 (2.1%) | 1031 (1.9%) | 4100 (2.9%) | 691 (2.6%) |
| CCS Category 237 | Ancillary Services | 205178 (71.5%) | 35665 (66.2%) | 107413 (77.1%) | 19831 (73.7%) |
| CCS Category 239 | Transportation - patient, provider, equipment | 648 (0.2%) | 62 (0.1%) | 419 (0.3%) | 51 (0.2%) |
| CCS Category 240 | Medications (Injections, infusions and other forms) | 30983 (10.8%) | 4596 (8.5%) | 17922 (12.9%) | 2876 (10.7%) |
| CCS Category 241 | Visual aids and other optical supplies | 1158 (0.4%) | 115 (0.2%) | 550 (0.4%) | 55 (0.2%) |
| CCS Category 242 | Hearing devices and audiology supplies | 16214 (5.6%) | 3170 (5.9%) | 8494 (6.1%) | 1792 (6.7%) |
| CCS Category 243 | DME and supplies | 11105 (3.9%) | 1816 (3.4%) | 7055 (5.1%) | 1209 (4.5%) |
| CCS Category 244 | Gastric bypass and volume reduction | 48 (0.0%) | 9 (0.0%) | 22 (0.0%) | 5 (0.0%) |
| Hierarchical Condition Categories (HCC) v24 for Chronic Medical Conditions | | | | | |
| HCC Category 1 | HIV/AIDS | 2438 (0.8%) | 513 (1.0%) | 1248 (0.9%) | 236 (0.9%) |
| HCC Category 2 | Septicemia, Sepsis, Systemic Inflammatory Response Syndrome/Shock | 34262 (11.9%) | 7128 (13.2%) | 24416 (17.5%) | 5138 (19.1%) |
| HCC Category 6 | Opportunistic Infections | 2001 (0.7%) | 408 (0.8%) | 1879 (1.3%) | 374 (1.4%) |
| HCC Category 8 | Metastatic Cancer and Acute Leukemia | 9274 (3.2%) | 1899 (3.5%) | 7036 (5.0%) | 1382 (5.1%) |
| HCC Category 9 | Lung and Other Severe Cancers | 10451 (3.6%) | 1983 (3.7%) | 7867 (5.6%) | 1536 (5.7%) |
| HCC Category 10 | Lymphoma and Other Cancers | 7162 (2.5%) | 1556 (2.9%) | 4673 (3.4%) | 980 (3.6%) |
| HCC Category 11 | Colorectal, Bladder, and Other Cancers | 16885 (5.9%) | 3572 (6.6%) | 12306 (8.8%) | 2546 (9.5%) |
| HCC Category 12 | Breast, Prostate, and Other Cancers and Tumors | 31755 (11.1%) | 7138 (13.2%) | 18956 (13.6%) | 4238 (15.8%) |
| HCC Category 17 | Diabetes with Acute Complications | 1959 (0.7%) | 414 (0.8%) | 1780 (1.3%) | 415 (1.5%) |
| HCC Category 18 | Diabetes with Chronic Complications | 80432 (28.0%) | 16517 (30.6%) | 52561 (37.7%) | 11187 (41.6%) |
| HCC Category 19 | Diabetes without Complication | 97806 (34.1%) | 20064 (37.2%) | 58897 (42.3%) | 12512 (46.5%) |
| HCC Category 21 | Protein-Calorie Malnutrition | 16643 (5.8%) | 3585 (6.6%) | 13421 (9.6%) | 2880 (10.7%) |
| HCC Category 22 | Morbid Obesity | 18780 (6.5%) | 4098 (7.6%) | 9906 (7.1%) | 2081 (7.7%) |
| HCC Category 23 | Other Significant Endocrine and Metabolic Disorders | 14998 (5.2%) | 3698 (6.9%) | 10111 (7.3%) | 2433 (9.0%) |
| HCC Category 27 | End-Stage Liver Disease | 3904 (1.4%) | 676 (1.3%) | 2699 (1.9%) | 516 (1.9%) |
| HCC Category 28 | Cirrhosis of Liver | 9432 (3.3%) | 1781 (3.3%) | 5936 (4.3%) | 1290 (4.8%) |
| HCC Category 29 | Chronic Hepatitis | 8785 (3.1%) | 1323 (2.5%) | 5154 (3.7%) | 776 (2.9%) |
| HCC Category 33 | Intestinal Obstruction/Perforation | 11726 (4.1%) | 2436 (4.5%) | 8441 (6.1%) | 1674 (6.2%) |
| HCC Category 34 | Chronic Pancreatitis | 2128 (0.7%) | 426 (0.8%) | 1550 (1.1%) | 319 (1.2%) |
| HCC Category 35 | Inflammatory Bowel Disease | 4504 (1.6%) | 895 (1.7%) | 2641 (1.9%) | 533 (2.0%) |
| HCC Category 39 | Bone/Joint/Muscle Infections/Necrosis | 12529 (4.4%) | 2671 (5.0%) | 10526 (7.6%) | 2238 (8.3%) |
| HCC Category 40 | Rheumatoid Arthritis and Inflammatory Connective Tissue Disease | 12431 (4.3%) | 3050 (5.7%) | 6071 (4.4%) | 1451 (5.4%) |
| HCC Category 46 | Severe Hematological Disorders | 2062 (0.7%) | 347 (0.6%) | 1496 (1.1%) | 235 (0.9%) |
| HCC Category 47 | Disorders of Immunity | 6393 (2.2%) | 1306 (2.4%) | 4527 (3.2%) | 929 (3.5%) |
| HCC Category 48 | Coagulation Defects and Other Specified Hematological Disorders | 18414 (6.4%) | 3692 (6.8%) | 12586 (9.0%) | 2493 (9.3%) |
| HCC Category 54 | Drug/Alcohol Psychosis | 2110 (0.7%) | 342 (0.6%) | 1229 (0.9%) | 202 (0.8%) |
| HCC Category 55 | Drug/Alcohol Dependence | 26986 (9.4%) | 5089 (9.4%) | 14428 (10.4%) | 2825 (10.5%) |
| HCC Category 56 | Major Depressive, Bipolar, and Paranoid Disorders | 6786 (2.4%) | 1302 (2.4%) | 3696 (2.7%) | 768 (2.9%) |
| HCC Category 57 | Schizophrenia | 7256 (2.5%) | 1319 (2.4%) | 4203 (3.0%) | 815 (3.0%) |
| HCC Category 58 | Reactive and Unspecified Psychosis | 2949 (1.0%) | 513 (1.0%) | 1538 (1.1%) | 303 (1.1%) |
| HCC Category 59 | Personality Disorders | 72541 (25.3%) | 14794 (27.4%) | 33505 (24.0%) | 6990 (26.0%) |
| HCC Category 60 | Eating Disorders | 6013 (2.1%) | 1208 (2.2%) | 2579 (1.9%) | 521 (1.9%) |
| HCC Category 70 | Quadriplegia | 6695 (2.3%) | 1549 (2.9%) | 5871 (4.2%) | 1273 (4.7%) |
| HCC Category 71 | Paraplegia | 7427 (2.6%) | 1900 (3.5%) | 6103 (4.4%) | 1439 (5.3%) |
| HCC Category 72 | Spinal Cord Disorders/Injuries | 7826 (2.7%) | 2004 (3.7%) | 5909 (4.2%) | 1409 (5.2%) |
| HCC Category 73 | Amyotrophic Lateral Sclerosis and Other Motor Neuron Disease | 684 (0.2%) | 111 (0.2%) | 712 (0.5%) | 111 (0.4%) |
| HCC Category 74 | Cerebral Palsy | 107 (0.0%) | 23 (0.0%) | 85 (0.1%) | 17 (0.1%) |
| HCC Category 75 | Myasthenia Gravis/Myoneural Disorders, Inflammatory and Toxic Neuropathy | 3148 (1.1%) | 780 (1.4%) | 2171 (1.6%) | 554 (2.1%) |
| HCC Category 76 | Muscular Dystrophy | 71 (0.0%) | 9 (0.0%) | 50 (0.0%) | 6 (0.0%) |
| HCC Category 77 | Multiple Sclerosis | 3378 (1.2%) | 812 (1.5%) | 2131 (1.5%) | 451 (1.7%) |
| HCC Category 78 | Parkinson's and Huntington's Diseases | 7247 (2.5%) | 1293 (2.4%) | 4467 (3.2%) | 830 (3.1%) |
| HCC Category 79 | Seizure Disorders and Convulsions | 10365 (3.6%) | 2074 (3.8%) | 6674 (4.8%) | 1396 (5.2%) |
| HCC Category 80 | Coma, Brain Compression/Anoxic Damage | 862 (0.3%) | 163 (0.3%) | 825 (0.6%) | 136 (0.5%) |
| HCC Category 82 | Respirator Dependence/Tracheostomy Status | 2543 (0.9%) | 403 (0.7%) | 2899 (2.1%) | 487 (1.8%) |
| HCC Category 83 | Respiratory Arrest | 131 (0.0%) | 16 (0.0%) | 150 (0.1%) | 19 (0.1%) |
| HCC Category 84 | Cardio-Respiratory Failure and Shock | 23491 (8.2%) | 4931 (9.1%) | 19757 (14.2%) | 4037 (15.0%) |
| HCC Category 85 | Congestive Heart Failure | 47450 (16.5%) | 10028 (18.6%) | 33173 (23.8%) | 7195 (26.7%) |
| HCC Category 86 | Acute Myocardial Infarction | 10418 (3.6%) | 2301 (4.3%) | 7710 (5.5%) | 1667 (6.2%) |
| HCC Category 87 | Unstable Angina and Other Acute Ischemic Heart Disease | 5776 (2.0%) | 941 (1.7%) | 4158 (3.0%) | 646 (2.4%) |
| HCC Category 88 | Angina Pectoris | 14212 (5.0%) | 2842 (5.3%) | 9081 (6.5%) | 1801 (6.7%) |
| HCC Category 96 | Specified Heart Arrhythmias | 50974 (17.8%) | 10575 (19.6%) | 33032 (23.7%) | 6767 (25.1%) |
| HCC Category 99 | Cerebral Hemorrhage | 2136 (0.7%) | 442 (0.8%) | 1430 (1.0%) | 334 (1.2%) |
| HCC Category 100 | Ischemic or Unspecified Stroke | 16749 (5.8%) | 3596 (6.7%) | 11067 (7.9%) | 2408 (8.9%) |
| HCC Category 103 | Hemiplegia/Hemiparesis | 8320 (2.9%) | 1719 (3.2%) | 5959 (4.3%) | 1238 (4.6%) |
| HCC Category 104 | Monoplegia, Other Paralytic Syndromes | 1400 (0.5%) | 328 (0.6%) | 1040 (0.7%) | 217 (0.8%) |
| HCC Category 106 | Atherosclerosis of the Extremities with Ulceration or Gangrene | 6179 (2.2%) | 1178 (2.2%) | 5627 (4.0%) | 1101 (4.1%) |
| HCC Category 107 | Vascular Disease with Complications | 12202 (4.3%) | 2572 (4.8%) | 9040 (6.5%) | 1931 (7.2%) |
| HCC Category 108 | Vascular Disease | 49079 (17.1%) | 9919 (18.4%) | 33791 (24.2%) | 6818 (25.3%) |
| HCC Category 111 | Chronic Obstructive Pulmonary Disease | 56516 (19.7%) | 11001 (20.4%) | 36180 (26.0%) | 7026 (26.1%) |
| HCC Category 112 | Fibrosis of Lung and Other Chronic Lung Disorders | 7160 (2.5%) | 1798 (3.3%) | 4988 (3.6%) | 1319 (4.9%) |
| HCC Category 114 | Aspiration and Specified Bacterial Pneumonias | 8434 (2.9%) | 1676 (3.1%) | 9016 (6.5%) | 1769 (6.6%) |
| HCC Category 115 | Pneumococcal Pneumonia, Empyema, Lung Abscess | 3772 (1.3%) | 674 (1.3%) | 3307 (2.4%) | 656 (2.4%) |
| HCC Category 122 | Proliferative Diabetic Retinopathy and Vitreous Hemorrhage | 2904 (1.0%) | 586 (1.1%) | 2302 (1.7%) | 486 (1.8%) |
| HCC Category 124 | Exudative Macular Degeneration | 3220 (1.1%) | 770 (1.4%) | 1860 (1.3%) | 426 (1.6%) |
| HCC Category 134 | Dialysis Status | 3849 (1.3%) | 665 (1.2%) | 3515 (2.5%) | 627 (2.3%) |
| HCC Category 135 | Acute Renal Failure | 43033 (15.0%) | 8721 (16.2%) | 32505 (23.3%) | 6564 (24.4%) |
| HCC Category 136 | Chronic Kidney Disease, Stage 5 | 6786 (2.4%) | 1176 (2.2%) | 5855 (4.2%) | 1081 (4.0%) |
| HCC Category 137 | Chronic Kidney Disease, Severe (Stage 4) | 9515 (3.3%) | 2152 (4.0%) | 7656 (5.5%) | 1718 (6.4%) |
| HCC Category 138 | Chronic Kidney Disease, Moderate (Stage 3) | 22285 (7.8%) | 659 (1.2%) | 14832 (10.6%) | 335 (1.2%) |
| HCC Category 157 | Pressure Ulcer of Skin with Necrosis Through to Muscle, Tendon, or Bone | 4491 (1.6%) | 1055 (2.0%) | 3705 (2.7%) | 830 (3.1%) |
| HCC Category 158 | Pressure Ulcer of Skin with Full Thickness Skin Loss | 9515 (3.3%) | 2040 (3.8%) | 7739 (5.6%) | 1733 (6.4%) |
| HCC Category 161 | Chronic Ulcer of Skin, Except Pressure | 19601 (6.8%) | 3873 (7.2%) | 16299 (11.7%) | 3322 (12.3%) |
| HCC Category 162 | Severe Skin Burn or Condition | 89 (0.0%) | 21 (0.0%) | 68 (0.0%) | 16 (0.1%) |
| HCC Category 166 | Severe Head Injury | 85 (0.0%) | 12 (0.0%) | 72 (0.1%) | 10 (0.0%) |
| HCC Category 167 | Major Head Injury | 2719 (0.9%) | 564 (1.0%) | 1635 (1.2%) | 314 (1.2%) |
| HCC Category 169 | Vertebral Fractures without Spinal Cord Injury | 2980 (1.0%) | 808 (1.5%) | 1932 (1.4%) | 503 (1.9%) |
| HCC Category 170 | Hip Fracture/Dislocation | 3146 (1.1%) | 656 (1.2%) | 2000 (1.4%) | 434 (1.6%) |
| HCC Category 173 | Traumatic Amputations and Complications | 1203 (0.4%) | 235 (0.4%) | 1049 (0.8%) | 202 (0.8%) |
| HCC Category 176 | Complications of Specified Implanted Device or Graft | 15745 (5.5%) | 3937 (7.3%) | 13429 (9.6%) | 3239 (12.0%) |
| HCC Category 186 | Major Organ Transplant or Replacement Status | 1056 (0.4%) | 206 (0.4%) | 814 (0.6%) | 178 (0.7%) |
| HCC Category 188 | Artificial Openings for Feeding or Elimination | 11345 (4.0%) | 2419 (4.5%) | 10268 (7.4%) | 2219 (8.2%) |
| HCC Category 189 | Amputation Status, Lower Limb/Amputation Complications | 8375 (2.9%) | 1754 (3.3%) | 7209 (5.2%) | 1505 (5.6%) |

Abbreviations: AIDS: Acquired Immunodeficiency Syndrome; CCS: Clinical Classification Software; CNS: Central Nervous System; CT: Computed Tomography; GI: Gastrointestinal; HCC: Hierarchical Clinical Categories; HIV: Human Immunodeficiency Virus; OR: Operating Room
