# Supplementary material for: Personalized Antibiogram: A Novel Multitask Machine Learning Framework for Simultaneous Prediction of Antimicrobial Resistance Profile With Enhanced Detection of Carbapenem Resistance in Enterobacteriaceae
Source: Clin Infect Dis. 2026 Jan 17;83(1):e1–9. doi: 10.1093/cid/ciag027 (PMC13393128; doi:10.1093/cid/ciag027)
Supplement: ciag027_Supplementary_Data [file ciag027_supplementary_data.zip › Supplementary Table 3 20260106.docx]

**Supplementary Table 3.** Comparative Performance of Hospital Antibiograms, Single-Target, and Multi-Task Models by Antibiotic and Organism

| **Antibiotic** | **Prevalence** | **AUC** | | | **AUPRC** | | | **Sensitivity** | | | **Specificity** | | | **PPV** | | | **NPV** | | | **F1** | | | **Brier Score** | | |
| --- | --- | --- | --- | --- | --- | --- | --- | --- | --- | --- | --- | --- | --- | --- | --- | --- | --- | --- | --- | --- | --- | --- | --- | --- | --- |
|  |  | **M1** | **M2** | **M3** | **M1** | **M2** | **M3** | **M1** | **M2** | **M3** | **M1** | **M2** | **M3** | **M1** | **M2** | **M3** | **M1** | **M2** | **M3** | **M1** | **M2** | **M3** | **M1** | **M2** | **M3** |
| *Escherichia coli* | | | | | | | | | | | | | | | | | | | | | | | | | |
| Overall | 0.224 | 0.569 | 0.858 | 0.862 | 0.270 | 0.672 | 0.679 | 0.437 | 0.594 | 0.590 | 0.670 | 0.770 | 0.771 | 0.277 | 0.427 | 0.427 | 0.805 | 0.868 | 0.867 | 0.339 | 0.497 | 0.495 | 0.240 | 0.116 | 0.115 |
| Aminopenicillins | 0.479 | 0.563 | 0.747 | 0.753 | 0.537 | 0.750 | 0.757 | 0.445 | 0.562 | 0.567 | 0.646 | 0.783 | 0.794 | 0.536 | 0.704 | 0.717 | 0.559 | 0.661 | 0.666 | 0.486 | 0.625 | 0.633 | 0.246 | 0.200 | 0.198 |
| NS Cephalosporins | 0.262 | 0.620 | 0.774 | 0.786 | 0.359 | 0.628 | 0.638 | 0.642 | 0.682 | 0.623 | 0.535 | 0.712 | 0.794 | 0.328 | 0.457 | 0.518 | 0.808 | 0.863 | 0.856 | 0.434 | 0.547 | 0.566 | 0.231 | 0.148 | 0.145 |
| TMP/SMX | 0.241 | 0.573 | 0.759 | 0.765 | 0.292 | 0.577 | 0.585 | 0.488 | 0.549 | 0.565 | 0.615 | 0.821 | 0.816 | 0.287 | 0.494 | 0.494 | 0.791 | 0.851 | 0.855 | 0.362 | 0.520 | 0.527 | 0.239 | 0.145 | 0.143 |
| Fluoroquinolones | 0.310 | 0.591 | 0.812 | 0.818 | 0.385 | 0.721 | 0.727 | 0.374 | 0.649 | 0.625 | 0.744 | 0.827 | 0.859 | 0.396 | 0.627 | 0.665 | 0.726 | 0.840 | 0.836 | 0.385 | 0.638 | 0.644 | 0.233 | 0.146 | 0.144 |
| Aminopenicillin/  BLI Combinations | 0.363 | 0.568 | 0.737 | 0.743 | 0.416 | 0.645 | 0.653 | 0.259 | 0.529 | 0.548 | 0.813 | 0.796 | 0.786 | 0.441 | 0.597 | 0.593 | 0.658 | 0.748 | 0.753 | 0.327 | 0.561 | 0.570 | 0.240 | 0.190 | 0.188 |
| ES Cephalosporins | 0.129 | 0.606 | 0.822 | 0.825 | 0.187 | 0.578 | 0.585 | 0.604 | 0.679 | 0.685 | 0.528 | 0.804 | 0.803 | 0.160 | 0.340 | 0.341 | 0.900 | 0.944 | 0.945 | 0.253 | 0.453 | 0.455 | 0.260 | 0.078 | 0.078 |
| Antipseudomonal/  BLI Combinations | 0.040 | 0.613 | 0.753 | 0.762 | 0.072 | 0.213 | 0.231 | 0.288 | 0.578 | 0.586 | 0.863 | 0.787 | 0.807 | 0.081 | 0.102 | 0.112 | 0.967 | 0.978 | 0.979 | 0.126 | 0.173 | 0.188 | 0.225 | 0.035 | 0.034 |
| Carbapenems | 0.003 | 0.651 | 0.764 | 0.783 | 0.005 | 0.015 | 0.029 | 0.601 | 0.712 | 0.783 | 0.624 | 0.666 | 0.587 | 0.004 | 0.006 | 0.005 | 0.998 | 0.999 | 0.999 | 0.008 | 0.011 | 0.010 | 0.244 | 0.003 | 0.003 |
| *Klebsiella* spp. | | | | | | | | | | | | | | | | | | | | | | | | | |
| Overall | 0.147 | 0.651 | 0.846 | 0.848 | 0.232 | 0.585 | 0.586 | 0.650 | 0.671 | 0.680 | 0.556 | 0.786 | 0.789 | 0.201 | 0.350 | 0.356 | 0.902 | 0.933 | 0.935 | 0.307 | 0.460 | 0.467 | 0.237 | 0.088 | 0.088 |
| NS Cephalosporins | 0.268 | 0.596 | 0.781 | 0.781 | 0.350 | 0.632 | 0.627 | 0.802 | 0.686 | 0.681 | 0.306 | 0.727 | 0.738 | 0.297 | 0.479 | 0.487 | 0.809 | 0.863 | 0.863 | 0.434 | 0.564 | 0.568 | 0.248 | 0.149 | 0.150 |
| TMP/SMX | 0.152 | 0.584 | 0.816 | 0.821 | 0.203 | 0.584 | 0.587 | 0.511 | 0.687 | 0.694 | 0.604 | 0.796 | 0.800 | 0.188 | 0.376 | 0.383 | 0.874 | 0.934 | 0.936 | 0.274 | 0.486 | 0.493 | 0.246 | 0.091 | 0.091 |
| Fluoroquinolones | 0.166 | 0.586 | 0.808 | 0.812 | 0.212 | 0.583 | 0.588 | 0.751 | 0.700 | 0.694 | 0.365 | 0.768 | 0.776 | 0.191 | 0.376 | 0.382 | 0.880 | 0.928 | 0.927 | 0.305 | 0.489 | 0.493 | 0.256 | 0.101 | 0.101 |
| Aminopenicillin/  BLI Combinations | 0.248 | 0.588 | 0.760 | 0.763 | 0.348 | 0.594 | 0.596 | 0.553 | 0.611 | 0.635 | 0.548 | 0.769 | 0.752 | 0.287 | 0.465 | 0.457 | 0.788 | 0.857 | 0.862 | 0.378 | 0.528 | 0.531 | 0.244 | 0.146 | 0.145 |
| ES Cephalosporins | 0.128 | 0.600 | 0.840 | 0.844 | 0.183 | 0.593 | 0.598 | 0.700 | 0.703 | 0.734 | 0.418 | 0.816 | 0.793 | 0.150 | 0.360 | 0.343 | 0.905 | 0.949 | 0.953 | 0.248 | 0.476 | 0.467 | 0.259 | 0.076 | 0.076 |
| Antipseudomonal/  BLI Combinations | 0.069 | 0.605 | 0.794 | 0.800 | 0.101 | 0.364 | 0.368 | 0.449 | 0.646 | 0.659 | 0.716 | 0.795 | 0.791 | 0.105 | 0.190 | 0.190 | 0.946 | 0.968 | 0.969 | 0.170 | 0.293 | 0.294 | 0.233 | 0.053 | 0.053 |
| Carbapenems | 0.014 | 0.721 | 0.839 | 0.847 | 0.055 | 0.230 | 0.245 | 0.518 | 0.715 | 0.709 | 0.809 | 0.805 | 0.842 | 0.036 | 0.048 | 0.058 | 0.992 | 0.995 | 0.995 | 0.068 | 0.090 | 0.108 | 0.175 | 0.012 | 0.012 |

Abbreviations: AUC: area under the receiver operating characteristic curve; AUPRC: area under the precision-recall curve; NS: narrow-spectrum; ES: extended-spectrum; BLI: beta-lactamase inhibitor; TMP/SMX: trimethoprim/sulfamethoxazole; F1: F1 score; NPV: negative predictive value; PPV: positive predictive value; M1: hospital antibiogram-only logistic regression baseline model; M2: single-target XGBoost model; M3: multi-task XGBoost model.
