# Supplementary material for: Personalized Antibiogram: A Novel Multitask Machine Learning Framework for Simultaneous Prediction of Antimicrobial Resistance Profile With Enhanced Detection of Carbapenem Resistance in Enterobacteriaceae
Source: Clin Infect Dis. 2026 Jan 17;83(1):e1–9. doi: 10.1093/cid/ciag027 (PMC13393128; doi:10.1093/cid/ciag027)
Supplement: ciag027_Supplementary_Data [file ciag027_supplementary_data.zip › Technical Supplement 20260106.docx]

***Technical Supplement***

(Please see the last page for the list of abbreviations)

1. **Dataset Preparation and Long-format Transformation**

We converted the antibiogram table (one row per isolate with many antibiotic outcomes) into a long table where each row is (isolate $i$, antibiotic $a$) with a binary label $yᵢ,ₐ\in0\left( susceptible \right),1\left( nonsusceptible \right)$. Rows where *a* were untested were dropped. We appended a categorical variable $task\_id=a$ to the predictors and trained a single booster $f\left( x,task\_id \right)$ that outputs $P\left( y=1 | x,task\_id \right)$. This is a problem‑transformation approach to multi-task learning: trees can split directly on task_id to learn task-specific substructures while sharing upstream partitions across related antibiotics.

The final long-format dataset comprised approximately 4.66 million rows for *E. coli* and 1.89 million rows for *Klebsiella spp.*, with each row corresponding to a single antibiotic resistance prediction task. This included data from 2017 through 2024, from 127 VHA hospitals. The final tasks included resistance predictions to eight clinically important antimicrobial classes: aminopenicillins, NS cephalosporins, TMP/SMX, FQ, aminopenicillin/BLI combinations, ES cephalosporins, antipseudomonal penicillin/BLI combinations, and carbapenems.

1. **Group-Aware Cross-Validation**

A critical methodological step in our model development was the implementation of patient-aware (group-aware) CV. To ensure rigorous evaluation and to prevent patient-level data leakage, we employed a 5-fold group-wise cross-validation scheme using the patient identifier as the grouping variable. This method guarantees that data from the same patient did not appear in both the training and validation sets within any fold, which is crucial given the longitudinal nature of clinical microbiology data and repeated patient observations.

1. **Feature Engineering and Encoding**

We analyzed culture results in a long-format dataset where each row represents a single isolate × antibiotic-class outcome (“task”). Features that describe the patient, facility, and specimen are repeated across tasks for the same isolate. For each variable group, we have added a table that serves as a toy example to help understand the real data.

- 1. Core identifiers and timing
     1. Specimen_ID – Unique specimen/isolate identifier.
     2. Patient_ID – Patient identifier across the VHA system.
     3. Facility_ID – VA facility code.
     4. SpecimenDateTime – Datetime of specimen collection.

| **Specimen_ID** | **Patient_ID** | **Facility_ID** | **SpecimenDateTime** |
| --- | --- | --- | --- |
| 1 | 1 | 2 | 2021-06-03 12:00:00 |
| 2 | 2 | 22 | 2018-02-11 01:25:00 |
| 3 | 3 | 9 | 2018-07-24 15:35:00 |
| 4 | 4 | 8 | 2022-05-20 17:15:00 |
| 5 | 5 | 6 | 2019-08-08 09:21:00 |

- 1. Demographics
     1. Age – Age in years at collection.
     2. Gender – 1 = male, 0 = female.
     3. Urban_Rural – Rurality; U = urban, R = rural

| **Specimen_ID** | **Patient_ID** | **Gender** | **Age** | **Urban_Rural** |
| --- | --- | --- | --- | --- |
| 1 | 1 | 1 | 81 | U |
| 2 | 2 | 0 | 66 | U |
| 3 | 3 | 1 | 70 | R |
| 4 | 4 | 1 | 63 | U |
| 5 | 5 | 1 | 87 | U |

- 1. Specimen/site indicators (binary, 1/0)
     1. Specimen_Type – Clinical source of the specimen. Recategorized from multi-category to 3-levels: "Blood" → "Blood", "Urine" → "Urine", "Fecal/Rectal Swab", "Lower Respiratory Tract", and "Other" → "Other"

| **Specimen_ID** | **Patient_ID** | **Specimen_Type** |
| --- | --- | --- |
| 1 | 1 | Urine |
| 2 | 2 | Urine |
| 3 | 3 | Blood |
| 4 | 4 | Blood |
| 5 | 5 | Urine |

- 1. Facility context
     1. Complexity_Tier – Indicates the complexity level of a VHA facility. The levels are based on factors such as patient risk, teaching and research activities, and the number of patients. The levels are:

1a: Highest complexity with the most patients, highest risk, and extensive teaching and research. These facilities have the most advanced ICU units.

1b: Very high complexity with large numbers of patients, high risk, and significant teaching and research. These facilities have advanced ICU units.

1c: High complexity with many patients, high risk, and considerable teaching and research. These facilities have advanced ICU units.

2: Medium complexity with moderate patient numbers and risk, some teaching and research. These facilities have mid-level ICU units.

3: Low complexity with fewer patients, lower risk, and little or no teaching and research. These facilities have basic ICU units.

If there are multiple facility IDs within a station, we use the most common (mode) complexity level.

- - 1. Residency_Tier – Indicates the number of training positions for new doctors at a VA facility, divided into four levels (1, 2, 3, 4). If there are multiple entries for a facility, use the middle value. If any information is missing, it is set to 1, the lowest level.
    2. BedNum – The number of operating beds available at the facility. If there are multiple entries for the same facility, use the median value. If the number of beds is missing, it is set to 0.

| **Specimen_ID** | **Patient_ID** | **Facility_ID** | **Complexity_Tier** | **Residency_Tier** | **BedNum** |
| --- | --- | --- | --- | --- | --- |
| 1 | 1 | 2 | 1b | 3 | 226.0 |
| 2 | 2 | 22 | 1b | 3 | 167.0 |
| 3 | 3 | 9 | 1c | 3 | 96.0 |
| 4 | 4 | 8 | 1a | 4 | 518.0 |
| 5 | 5 | 6 | 1c | 3 | 184.0 |

- 1. Comorbidity and procedure flags (binary, 1/0)
     1. CCS_Cat* – Indicator variables for procedures, based on CCS. Each flag equals 1 if a patient had this procedure within 90 days prior to SpecimenDateTime, else 0.
     2. HCC_Cat* – Indicator variables for comorbidities, based on HCC. Each flag equals 1 if a patient was treated for this comorbidity within 5 years from SpecimenDateTime, else 0.

| **Specimen_ID** | **Patient_ID** | **CCS_Cat1** | **CCS_Cat2** | **…** | **HCC_Cat1** | **HCC_Cat2** | **…** |
| --- | --- | --- | --- | --- | --- | --- | --- |
| 1 | 1 | 0 | 0 | … | 0 | 1 | … |
| 2 | 2 | 0 | 0 | … | 0 | 0 | … |
| 3 | 3 | 0 | 0 | … | 0 | 0 | … |
| 4 | 4 | 0 | 0 | … | 0 | 0 | … |
| 5 | 5 | 0 | 0 | … | 0 | 1 | … |

- 1. History features: prior antibiotics and prior susceptibility
     1. Past antibiotic exposure (patient-level medication history)

For each antibiotic class, we compute cumulative days of exposure within pre-specified, non-overlapping look-back windows prior to SpecimenDateTime. Columns are named: ⟨Class⟩_1to3_days, ⟨Class⟩_4to7_days, ⟨Class⟩_8to14_days, ⟨Class⟩_15to30_days, ⟨Class⟩_31to90_days, ⟨Class⟩_91to365_days, ⟨Class⟩_GT365_days. A non-negative integer value represents the total number of days the patient received any agent in that class within the specified window. Examples: Fluoroquinolone_31to90_days = 7 ⇒ seven total exposure days in days 31–90 before collection. Amino_PCN_91to365_days = 10 ⇒ ten total exposure days in days 91–365 before collection. These “days-in-window” counts enable models to learn short-term vs. long-term selection pressure patterns without leaking future information.

- - 1. Past isolate susceptibility (patient-level and facility-level signals)

Patient-level last-result recency signal: For each class, we derived Last⟨Class⟩, which compresses the most recent pre-index susceptibility result and its recency into a single numeric feature. Let $d$ be the number of days between the current specimen collection and the most recent prior susceptibility result for that class.

$$Last\left\langle Class \right\rangle=\left\{ \begin{aligned} +\frac{1}{d}, if the last result was non-susceptible \\ -\frac{1}{d}, if the last result was susceptible \\ 0, if no prior result exists \end{aligned} \right.$$

We guarded against division by zero (same-day cases use $d\geq1$). The sign encodes direction (non-susceptible vs susceptible); the magnitude encodes recency (more recent ⇒ larger absolute value). 0 represents no prior signal, and not neutral susceptibility. Exposure windows were anchored strictly before SpecimenDateTime; no future information is used. If desired, Last⟨Class⟩ can be decomposed into two features, (i) last result class and (ii) days since last result, but the signed-reciprocal scalar performs well and keeps dimensionality low.

- - 1. Facility-year susceptibility proportion (SPR)

For each class, we included ⟨Class⟩_SPR, the share susceptible at the same facility in the prior calendar year (i.e., conventional hospital antibiogram values for each antimicrobial-hospital-year combination). ⟨Class⟩_SPR value ∈ [0, 1]. If a facility-year estimate is missing/sparse, we substitute the national prior-year proportion for that class.

| **Specimen_ID** | **Patient_ID** | **Sulfa_SPR** | **LastSulfa** | **Sulfa_1to3_days** | **Sulfa_4to7_days** | **…** | **Sulfa_91to365_days** | **Sulfa_GT365_days** |
| --- | --- | --- | --- | --- | --- | --- | --- | --- |
| 1 | 1 | 0.6605 | -0.000884 | 0 | 0 | … | 0 | 0 |
| 2 | 2 | 0.7727 | 0.000000 | 0 | 0 | … | 0 | 0 |
| 3 | 3 | 0.7620 | 0.000000 | 0 | 0 | … | 0 | 0 |
| 4 | 4 | 0.7933 | 0.000000 | 0 | 0 | … | 0 | 0 |
| 5 | 5 | 0.8077 | 0.000000 | 0 | 0 | … | 0 | 20 |

- 1. Outcome definition (per antibiotic class)
     1. Coding in wide format: 1 = tested & non-susceptible, 0 = tested & susceptible, -1 = not tested.
     2. Coding in long format: For each isolate and antibiotic class with a performed test, we keep one row with a single binary label y (1 = non-susceptible, 0 = susceptible) and a categorical task_id naming the class. Rows with -1 (not tested) are not included in the modeling table.

| **Specimen_ID** | **Patient_ID** | **Amino_PCN** | **NS_Ceph** | **TMP_SMX** | **FQ** | **Amino_PCN-BLI** | **ES_Ceph** | **Anti-PSA_PCN-BLI** | **Carbapenem** |
| --- | --- | --- | --- | --- | --- | --- | --- | --- | --- |
| 1 | 1 | 0 | -1 | 0 | 0 | 0 | 0 | 0 | 0 |
| 2 | 2 | 1 | 0 | 0 | 0 | 1 | 0 | -1 | 0 |
| 3 | 3 | 0 | 0 | 0 | 0 | 0 | 0 | 0 | 0 |
| 4 | 4 | 1 | 1 | 1 | 0 | 1 | 0 | 0 | 0 |
| 5 | 5 | 0 | 0 | 0 | 0 | 0 | 0 | 0 | 0 |

| **Specimen_ID** | **Patient_ID** | **task_id** | **label** |
| --- | --- | --- | --- |
| 1 | 1 | Amino_PCN | 0 |
| 1 | 1 | TMP_SMX | 0 |
| 1 | 1 | FQ | 0 |
| … | … | … | … |
| 5 | 5 | Anti-PSA_PCN-BLI | 0 |
| 5 | 5 | Carbapenem | 0 |

1. **Model Development: Hyperparameter Optimization**

To systematically identify optimal hyperparameter configurations for our unified XGBoost multi-task model, we performed extensive hyperparameter tuning using group-aware 5-fold cross-validation. Hyperparameters included:

- Maximum tree depth (max_depth): Controlling model complexity and risk of overfitting.
- Minimum child weight (min_child_weight): Preventing splits that result in very small leaves, mitigating overfitting and ensuring generalizable rules.
- Gamma (gamma): A split regularization term, requiring a minimum improvement in the loss function to justify additional splits.
- Row subsample (subsample): Introducing randomness and variance reduction through bagging.
- Learning rate (eta): Adjusting the step size of boosting iterations to balance convergence speed and performance.
- L2 regularization (reg_lambda) and L1 regularization (reg_alpha): Regularization techniques to reduce model complexity and prevent overfitting.

| **Hyperparameter** | **Values for *E. coli*** | **Values for *Klebsiella spp.*** |
| --- | --- | --- |
| Maximum tree depth (max_depth) | 4, 6, 8, 12 | 6, 10, 12, 15, 20 |
| Minimum child weight (min_child_weight) | 1, 5, 8, 10, 12, 16, 20, 25 | 1, 5, 8, 10, 15 |
| Gamma (gamma) | 0.0, 0.1, 0.2 | 0.0, 0.1, 0.2 |
| Row subsample (subsample) | 0.65, 0.80 | 0.65, 0.80, 1.00 |
| Learning rate (eta) | 0.05, 0.10, 0.30 | 0.03, 0.05, 0.30 |
| L2 regularization (reg_lambda) | 1, 3, 5, 10 | 0, 1, 5, 10 |
| L1 regularization (reg_alpha) | 0.0, 0.5 | 0.0, 0.5 |

We conducted iterative grid searches to determine the optimal hyperparameter values. Each hyperparameter set was evaluated using the average AUROC over the 5-fold CV. Hyperparameter configurations resulting in memory errors or overly complex trees (e.g., depth > 12 with high learning rates) were excluded or retried with adjusted values. Ultimately, the best-performing hyperparameter set was identified as:

| **Hyperparameter** | **Optimal Value for *E. coli*** | **Optimal Value for *Klebsiella spp.*** |
| --- | --- | --- |
| max_depth | 6 | 15 |
| min_child_weight | 10 | 15 |
| gamma | 0.1 | 0.0 |
| subsample | 0.80 | 1.00 |
| eta | 0.10 | 0.05 |
| reg_lambda | 1.0 | 5 |
| reg_alpha | 0.0 | 0.5 |

1. **Final Model Training**

After selecting the optimal hyperparameters via CV, we trained the final model on the complete training dataset (2017–2023). Training was performed using XGBoost’s GPU-accelerated gpu_hist method, enabling fast computation despite the large size of our dataset. We applied early stopping (50 rounds) during training to prevent overfitting, thus ensuring efficient utilization of resources and avoiding unnecessary model complexity.

1. **Evaluation Metrics**

Given our multi-task and highly imbalanced clinical setting, we utilized multiple evaluation metrics:

- Overall AUROC: To evaluate discriminative ability across all antibiotic outcomes in a pooled manner.
- Per-antibiotic AUROC: To measure predictive performance for each antibiotic separately, highlighting variability across tasks.

1. **Operating point selection (Youden’s J)**

For each antibiotic, the model outputs a probability of non-susceptibility $\hat{p}$. We converted probabilities to class labels using a threshold $t$: predict non-susceptible if $\hat{p}\geq t$. On validation data (out-of-fold predictions), we computed sensitivity $Se\left( t \right)=\frac{TP}{TP+FN}$ and specificity $Sp\left( t \right)=\frac{TN}{TN+FP}$ over a set of candidate thresholds (we used the unique predicted probabilities, which induces the standard stepwise ROC). From these curves, we selected three operating points per antibiotic:

- Balanced threshold (default). We chose the threshold $t^{*}$ that maximized the Youden index, $J\left( t \right)=Se\left( t \right)+Sp\left( t \right)-1$. $J$ is prevalence-invariant and equivalent to maximizing balanced accuracy $\frac{Se+Sp}{2}$, making it a natural default when false negatives and false positives are similarly undesirable.
- High-sensitivity threshold (early coverage). We selected the smallest threshold $t$ that achieves $Se\left( t \right)\geq0.95$. If multiple thresholds satisfy this, we kept the one with the highest specificity; any remaining ties are broken by the larger $J$. If no threshold reached 0.95 due to finite-sample steps in the ROC, we took the threshold with the highest attainable sensitivity and applied the same tiebreakers.
- High-specificity threshold (confirmatory). Symmetrically, we selected the largest threshold $t$ that achieved $Sp\left( t \right)\geq0.95$. If several satisfy this, we kept the one with the highest sensitivity; residual ties are broken by the larger $J$. If 0.95 was not attainable, we took the threshold with the highest attainable specificity and applied the same tiebreakers.

To ensure robustness, all three thresholds were computed within each validation fold and then aggregated across folds (we use the median threshold) to yield a single operating point per antibiotic. These fold-derived thresholds were then applied unchanged to the independent 2024 test set. We did not use a weighted-Youden objective; the high-sensitivity and high-specificity choices were strictly constraint-based selections.

1. **Baselines**
   1. Hospital-antibiogram logistic regression (SPR-only): This baseline mimics a conventional antibiogram by using the facility-year susceptible proportion (SPR) as the only predictor for each antibiotic. For each species-antibiotic pair, we constructed $X=\left[ SPR \right]$ and a binary outcome $y\in0 \left( susceptible \right),1 \left( non-susceptible \right)$. We used the same temporal split as the primary model (train/validate on 2017-2023, test on 2024). Performance was estimated with 5‑fold StratifiedKFold on the training period to maintain class balance within folds. We fit scikit‑learn’s LogisticRegression(solver="liblinear") with the default L2 penalty and C=1.0 to predict non‑susceptibility from the facility‑year susceptible proportion (SPR) only. No feature scaling was required. Performance was estimated with 5‑fold StratifiedKFold on 2017–2023, and when a hard decision was needed, we chose thresholds by maximizing Youden’s 𝐽 on out‑of‑fold predictions; otherwise, we report AUROC on the 2024 test set. Missing facility-year SPR values, if any, were imputed using the national prior-year SPR for that antibiotic class.
   2. Single-task XGBoost (one model per antibiotic): To isolate the benefit of multi-task sharing, we trained separate binary XGBoost models, one per antibiotic, using the same feature set and temporal split as the unified model (train/validate: 2017–2023; test: 2024). For each antibiotic, rows with unperformed tests were excluded, and we applied 5-fold GroupKFold with Patient_ID as the grouping variable to prevent patient-level leakage across folds. Tuned hyperparameters included max_depth, min_child_weight, gamma, subsample, eta (learning rate), reg_lambda (L2), and reg_alpha (L1). Within each CV fold we used early stopping (~50 rounds) to find the fold‑specific best iteration. For the final refit on the full 2017–2023 training set, we set the number of boosting rounds (a.k.a. num_boost_round / n_estimators) to the median of those fold‑wise best iterations and retrained with the chosen hyperparameters. Unless otherwise specified, we used tree_method="gpu_hist". Test-set performance was reported per antibiotic (e.g., AUROC); when required, binary operating points were derived from validation predictions using Youden’s J for consistency with the unified model.

**Abbreviation Glossary**

- Amino_PCN – aminopenicillins.
- Amino_PCN‑BLI – aminopenicillin/BLI (β‑lactamase inhibitor) combinations.
- Anti‑PSA_PCN‑BLI – antipseudomonal (Anti‑PSA) penicillin/BLI combinations.
- AUROC – Area Under the Receiver Operating Characteristic curve; reported overall and per‑antibiotic.
- C = 1.0 – the inverse of regularization strength (smaller C ⇒ stronger regularization).
- Carbapenem – carbapenems.
- CCS: Clinical Classification Software
- E. coli – Escherichia coli.
- Early stopping (50 rounds) – stop boosting if no metric improvement within 50 iterations; used during tuning/training.
- ES_Ceph – extended‑spectrum cephalosporins (ES = extended spectrum).
- FN – False Negative
- FP – False Positive
- FQ – fluoroquinolones.
- GroupKFold – scikit‑learn group‑wise CV used for single‑task baselines.
- HCC: Hierarchical Conditions Category
- ICU – intensive care unit (used in facility complexity tier descriptions).
- Klebsiella spp. – Klebsiella species; spp. = multiple species.
- L2 penalty (a.k.a. ridge) – adds a penalty proportional to the square of the coefficient(s), shrinking them toward zero to reduce overfitting.
- LogisticRegression – a linear classifier that models the log‑odds of the positive class as a linear function of the inputs.
- NS_Ceph – narrow‑spectrum cephalosporins (NS = narrow spectrum).
- ROC – Receiver Operating Characteristic
- solver="liblinear" – uses the LIBLINEAR optimizer; it’s reliable for small feature sets and supports L1/L2 penalties.
- StratifiedKFold – class‑balance‑preserving CV used for the SPR‑only baseline.
- TMP_SMX – trimethoprim/sulfamethoxazole.
- TN – True Negative
- TP – True Positive
- tree_method="gpu_hist" – GPU‑accelerated histogram algorithm for final training (unified model).
- VA – U.S. Department of Veterans Affairs.
- VHA – Veterans Health Administration.
